# A screening platform to monitor RNA processing and protein-RNA interactions in ribonuclease P uncovers a small molecule inhibitor

**DOI:** 10.1093/nar/gkz285

**Published:** 2019-04-18

**Authors:** Ezequiel-Alejandro Madrigal-Carrillo, Carlos-Alejandro Díaz-Tufinio, Hugo-Aníbal Santamaría-Suárez, Marcelino Arciniega, Alfredo Torres-Larios

**Affiliations:** 1 Department of Biochemistry and Structural Biology, Instituto de Fisiología Celular, Universidad Nacional Autónoma de México, Mexico City, Mexico; 2 Tecnologico de Monterrey, Escuela de Ingeniería y Ciencias, Mexico City, Mexico

## Abstract

Ribonucleoprotein (RNP) complexes and RNA-processing enzymes are attractive targets for antibiotic development owing to their central roles in microbial physiology. For many of these complexes, comprehensive strategies to identify inhibitors are either lacking or suffer from substantial technical limitations. Here, we describe an activity-binding-structure platform for bacterial ribonuclease P (RNase P), an essential RNP ribozyme involved in 5′ tRNA processing. A novel, real-time fluorescence-based assay was used to monitor RNase P activity and rapidly identify inhibitors using a mini-helix and a pre-tRNA-like bipartite substrate. Using the mini-helix substrate, we screened a library comprising 2560 compounds. Initial hits were then validated using pre-tRNA and the pre-tRNA-like substrate, which ultimately verified four compounds as inhibitors. Biolayer interferometry-based binding assays and molecular dynamics simulations were then used to characterize the interactions between each validated inhibitor and the P protein, P RNA and pre-tRNA. X-ray crystallographic studies subsequently elucidated the structure of the P protein bound to the most promising hit, purpurin, and revealed how this inhibitor adversely affects tRNA 5′ leader binding. This integrated platform affords improved structure-function studies of RNA processing enzymes and facilitates the discovery of novel regulators or inhibitors.

## INTRODUCTION

Regulatory RNAs, ribozymes, and RNA-protein complexes are appealing antibiotic targets due to their essential functions in microbial metabolism (1–3). This clinical importance is exemplified by the ribosome, which is currently the target of roughly 50% of known antibiotics (4). The focus of this work is Ribonuclease P (RNase P), the only ribozyme other than the ribosome that is present in all three domains of life (see (5–8) for some reviews). This essential ribonucleoprotein complex remains to be exploited as a target for much-needed novel antibacterial agents (9).

The composition of RNase P varies across the three domains of life (10) and therefore may afford high selectivity in drug targeting (11,12). While in archaea and eukaryotes, RNase P is comprised of one RNA subunit and four to ten proteins (13), in bacteria, this complex is formed by an RNA subunit (P RNA, 350–400 nucleotides, 110–125 kDa) and a single protein (P protein, ∼110 amino acids, 13 kDa) (14). In all species, the P RNA serves as the primary biocatalyst (15) for the cleavage of the 5′-leader sequence of pre-tRNAs during tRNA maturation (16). The P protein, on the other hand, binds the distal 5′-leader region of the pre-tRNA substrate, enhances the affinity of metal ions, and assists in product release (17–20). RNase P is dependent on divalent metal ions (Mg^2+^ is needed for proper folding and activity (21–23)) and *in vitro*, high concentrations of Mg^2+^ are sufficient to allow P RNA catalysis, even in the absence of the protein subunit (15,24,25).

There are several known inhibitors of RNase P (reviewed in (12,26,27)), with iriginol hexaacetate and methylene blue being the most recently discovered (26,28). However, to date, neither natural nor highly specific RNase P inhibitors have been identified. The dynamic and flexible nature of the RNase P complex may have hindered the evolution of specific binders (29) and moreover, the protein subunit has some features of intrinsically disordered proteins, which could hamper the stable binding of ligands (30). There also may be an inherent limitation for small molecules to compete with the protein for binding to the P RNA, depending on the relative affinity of the P RNA:P protein pair.

Monitoring RNase P activity has traditionally relied on using radiolabeled RNA substrates (31). In this method, radiolabeled RNA is mixed with RNase P. The reaction is then monitored by denaturing polyacrylamide gel electrophoresis (Urea-PAGE) followed by auto-radiography (31). Very recently, alternatives to measure the kinetics of RNase P on pre-tRNA substrates have emerged. A recent paper developed a fluorescence polarization-based activity assay to monitor RNase P activity on a full-length substrate (26).

To expand the repertoire of inhibitors that may serve as clinical leads as antibiotics, a robust platform for assessing RNase P activity in high throughput is essential. Current activity protocols are either inhibited by 1% (v/v) DMSO or not amenable to high-throughput screening. Additionally, validation methods to verify efficacious RNase P inhibition are lacking. Recognizing these shortcomings, we aimed to create improved methods for discovering small molecule inhibitors of RNase P. To accomplish this goal, we focused on the following strategy: (i) developing a novel method to measure real-time RNase P activity in solution; (ii) obtaining real-time measurements of the binding of the RNase P holoenzyme, P RNA and P protein to their native substrate and inhibitors and (iii) obtaining structural information, *in silico* and through X-ray crystallography, on the binding of potential inhibitors to the P protein.

In this work, we present an RNase P activity assay that exploits a previously reported minimal model substrate (pMini3bpUG, herein referred to as Minihelix or Mh) (32,33). This substrate utilizes a FRET mechanism in which the RNase P substrate couples both a 3′ fluorophore and a 5′ non-fluorescent quencher. Cleavage and release of the quencher molecule by RNase P enables the detection of enzymatic activity by measuring fluorescence emission over time, which is amenable for monitoring steady-state kinetics and for high-throughput screening assays.

We then implemented this method to assess a compound library of 2560 small molecules and found four compounds that inhibit RNase P activity. These inhibitors were effective in the presence of both a canonical pre-tRNA substrate and a novel pre-tRNA-like substrate (herein referred to as ‘bipartite pre-tRNA’) that is composed of two RNA oligonucleotides and monitors the reaction in an analogous way to the Mh substrate. To avoid the sensitivity of RNase P processing to organic solvents using the bipartite pre-tRNA substrate, we dissolved the hits in PEG 200 rather than DMSO. This procedure allowed us to validate the inhibitory properties of these molecules under varied conditions.

Positive hits were then verified and characterized using biolayer interferometry (34), which allowed us to perform the following tasks: (i) define the affinity parameters of the RNase P holoenzyme, P RNA and P protein to the RNA substrates, (ii) discriminate between the interactions of a given compound with the holoenzyme, P RNA, P protein or the substrate and (iii) determine if a given compound hinders the binding of the holoenzyme to pre-tRNA or the P protein to the 5′-leader.

Once validated, we performed docking and molecular dynamics simulations with each hit and identified putative binding sites for two inhibitors on the P protein. Furthermore, purpurin, a competitive inhibitor which behaved as the most consistent hit across our assays, was shown to bind the P protein by X-ray crystallography, with its binding site corresponding to part of the 5′-leader binding site.

## MATERIALS AND METHODS

### Selection of bacterial RNase P

We chose to work with RNase P from the thermophilic bacterium *Thermotoga maritima* for several reasons. First, it represents the ancestral and most common ‘type A’ ribozyme, the same that is present in *Escherichia coli* (35). Second, the crystal structure of the RNase P holoenzyme from *T. maritima* in complex with tRNA (19) as well as the apo-structure of the RNA subunit (36) are known, thus facilitating structure-function studies. Third, the folding protocol of the RNase P holoenzyme from *T. maritima* (described below) leads to crystals that present a functional and homogeneous molecular complex (19). Fourth, although the RNase P from *T. maritima* has optimal activity at 50°C (37), the holoenzyme is active in a temperature range of 20–55°C.

### RNase P production

The preparation of P RNA and pre-tRNA from *T. maritima* was carried out by *in vitro* transcription and purified by denaturing 6% urea-PAGE with standard protocols as described previously (19,38). The P protein was purified according to (39). The A260/A280 ratio was between 0.6 and 0.7 for each preparation of purified protein.

### RNase P reconstitution

The reconstitution of the RNase P holoenzyme was performed as described in (40,19). This protocol produces a homogeneous and functional ribozyme that can be crystallized by mixing P RNA and P protein in a 1:1.1 molar ratio in 1× TH buffer (33 mM Tris–HCl, 66 mM HEPES, pH 7.4) and 400 mM ammonium acetate (AmOAc). The mixture was heated to 95°C for 2 min in a water bath, then chilled on ice for 2 min, after which MgCl_2_ was added to a final concentration of 100 mM. Although RNase P from *T. maritima* is catalytically active at 10 mM MgCl_2_ (19), we found that for our minihelix substrate, 100 mM MgCl_2_ demonstrated more robust activity. This higher concentration was used in all experiments described herein. The holoenzyme was further incubated at 50°C for 10 min and 37°C for 40 min. The final concentration of holoenzyme was 333 nM (35.6 μg/ml of P RNA and 4.7 μg/ml of P protein).

### Substrate minihelix (Mh)

**Figure 1.**
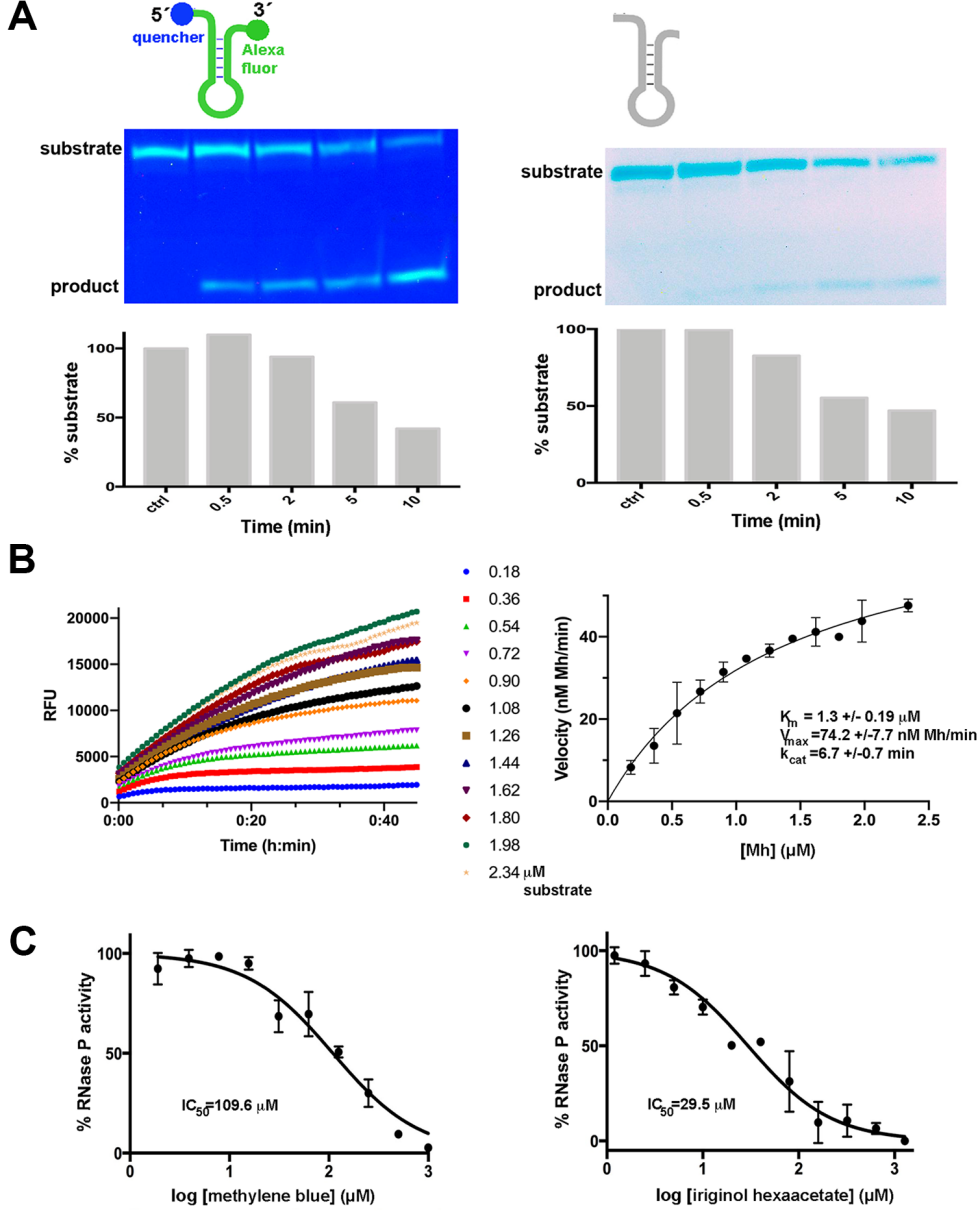
Validation of the fluorescent minihelix (Mh) substrate. (**A**) Cleavage by bacterial RNase P. Top. Schematic representation of the 24-nucleotide fluorescent (left) or non-fluorescent (right) minihelix (Mh) substrate with the predicted secondary structure according to Brannvall et al. (32). Middle left. RNase P holoenzyme from *Thermotoga maritima* cleaves the fluorescent Mh substrate, as visualized by 20% urea-PAGE under UV light. The fluorescence detected from both the substrate (upper band) and the product (lower band) is due to the unfolding of the Mh probe under the denaturing gel conditions. The time-dependent disappearance at 55°C of the 24 nt Mh fluorescent substrate (5 ng), with a simultaneous accumulation of the 15 nt product, confirms the catalytic cleavage of RNase P on the proposed minihelix. Middle right. FRET-pair addition does not affect the cleavage of RNase P on the fluorescent probe as visualized by UV after PAGE and ethidium bromide staining. Bottom. Similar time courses of substrate conversion are observed for the fluorescent (left) and non-fluorescent (right) Mh substrates. (**B**) Kinetic assays of RNase P with the fluorescent substrate show a typical Michaelis-Menten enzymatic behavior. Left. Representative plots of kinetic assays are shown. RNase P holoenzyme was incubated at 20°C at 11 nM, with increasing Mh concentrations as indicated. Right. Michaelis-Menten model fitting (*R*^2^ = 0.9368). Initial velocities from three independent trials were plotted against substrate concentration; error bars indicate the standard error for each plot. (**C**) The reported RNase P inhibitors methylene blue and iriginol hexaacetate inhibit bacterial RNase P from *Thermotoga maritima*. Left. The inhibition of RNase P by methylene blue. Dose-response curve (*R*^2^ = 0.9543) calculated from the normalized slope values from the linear portions of the kinetic assays in triplicate shown in Supplementary Figure S5. The reported K_i_ value using a conventional pre-tRNA substrate and the RNA subunit of different bacterial RNase P′s is between 14 and 28 μM (28). Right. Inhibition of RNase P by iriginol hexaacetate. Dose–response curve (R^2^ = 0.9556) calculated from the normalized slope values from assays in triplicate. The reported IC_50_ value using a fluorescent pre-tRNA substrate is 0.8 μM for *Bacillus subtilis* RNase P (26).

The minihelix (Mh) substrate (Figure 1A) was adopted from a previously characterized RNase P substrate, pMini3bpUG (32,33) and coupled to either Black Hole Quencher 1 (BHQ1) or Black Hole Quencher 2 (BHQ2) at the 5′-end and Alexa Fluor 488 at the 3′-end. This probe was purchased either from Sigma-Aldrich or Integrated DNA Technologies (IDT). A requested scale of 0.05 mmol yielded between 6.3 and 20 nmol (57.5 to 181 μg, enough for 1300–4000 reactions depending on the experimental substrate concentration). Before use, the minihelix was diluted from a 100 μM stock (dissolved in water) to 3.6 μM in activity buffer H (33 mM Tris–HCl, 66 mM HEPES, pH 7.4, 400 mM AmOAc and 100 mM MgCl_2_).

### Substrate bipartite pre-tRNA

The bipartite pre-tRNA substrate derived from pre-tRNA^Phe^ from *T. maritima* (Supplementary Figure S13) was designed for the ease of chemical synthesis and conceived from the fact that the anticodon loop is not required for RNase P recognition (Reiter *et al.*). As for the Mh substrate, fluorescent bipartite pre-tRNA was coupled to Black Hole Quencher 1 (BHQ1) at the 5′-end of the 5′ oligo, and to Alexa Fluor 488 at the 3′-end of the 3′ oligo (see Supplementary Table S1). The probes were purchased from Integrated DNA Technologies (IDT). A requested scale of 250 nmol yielded 27.4 nmol (0.36 mg) for the 5′ oligo and 3.8 nmol (50 μg) for the 3′ fluorescent oligo. Before use, the probes were diluted from a 100 μM stock (dissolved in water) and assembled by mixing and heating to 3.6 μM in activity buffer L (Tris–HCl pH 7.4 33 mM, HEPES 66 mM, 100 mM AmOAc and 10 mM MgCl_2_).

### Fluorescence-based activity assay

Reactions were performed in a total volume of 20 μl. Pipetting was done by hand using multichannel pipettes. First, an 18 μl reaction mixture containing 16.5 μl of activity buffer (H for Mh or L supplemented with 10% PEG 200 for bipartite pre-tRNA) and 270 nM (Mh) or 135 nM (bipartite pre-tRNA) (1.5 or 0.7 μl of the 3.6 μM stock) of the fluorescent substrate probe was pre-incubated on ice for at least 5 m in a black polystyrene 384-well microtiter microplate with a flat transparent bottom (Corning ref 3540). The holoenzyme was then added at a final concentration of 33 nM (2 μl of a 333 nM stock) in 1× TH buffer and mixed. Kinetic activity was monitored in a Synergy MX Multimode Detector (BioTek) at 37°C with rapid agitation. At least 20 readings were taken per well every 15 s. The probe was excited at 485 nm, and its emission was monitored at 535 nm with an integration time of 1 s. From the plots of relative fluorescent units (RFU) versus time, linear regression was used to determine the slopes in the linear portion of the curves, using at least 12 data points to obtain initial velocities. Non-linear fitting to obtain the Michaelis–Menten parameters was performed using GraphPad Prism^®^ 6.01 software.

### Gel-based activity assay

For gel-based activity assays, reactions were prepared as described in the previous section (fluorescence activity assay), incubated for 1–15 min at 37–50°C, then loaded onto a denaturing 7 M urea–20% acrylamide gel in the case of the Mh substrate or 19% acrylamide (40% acrylamide/bisacrylamide 38.5:1.5) for the bipartite pre-tRNA substrate. The activity conditions for the gel reported in Figure 3A, to monitor the cleavage of the holoenzyme and P RNA, were under 100 mM AmOAc and 10 mM MgCl_2._ The gels (13 × 16 × 0.15 cm) were electrophoresed at 20 V/cm for 2–3 h. UV shadowing or ethidium bromide staining was used to visualize RNA cleavage. Detection of minihelix fluorescence was achieved without staining using a UV lamp with excitation at ∼254 nm.

### Library screening

**Figure 2.**
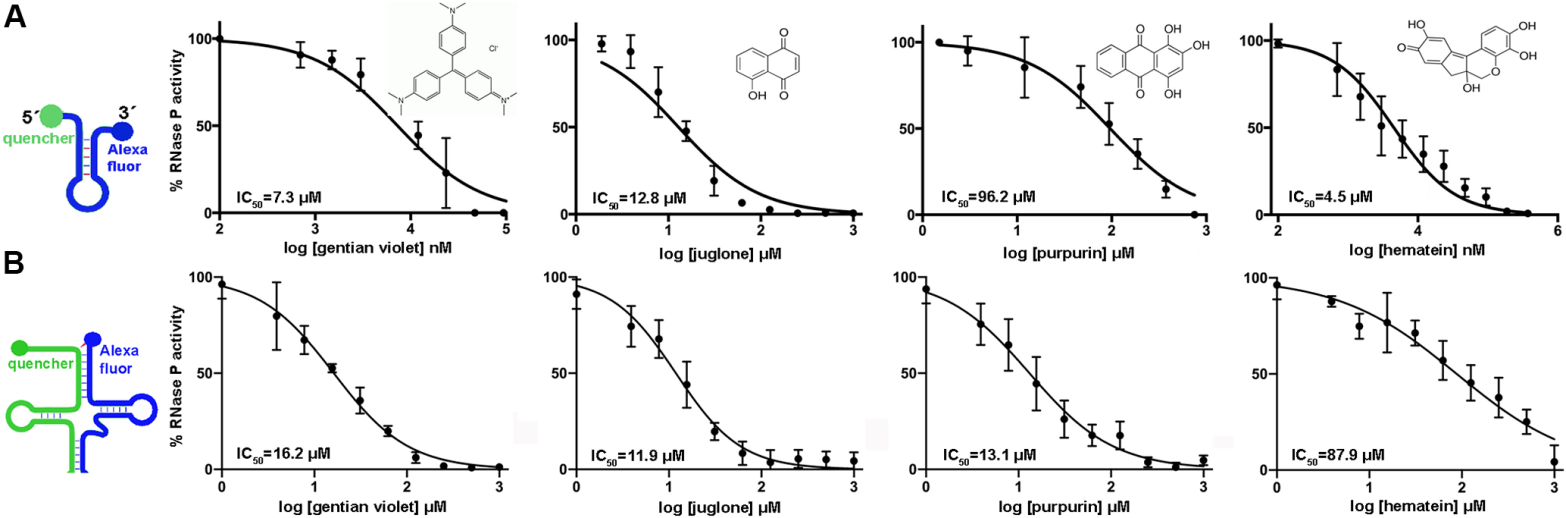
Four validated hits inhibit RNase P holoenzyme from *Thermotoga maritima* in the micromolar range using the substrates Mh and bipartite pre-tRNA. After the screening step (Supplementary Figures S6–S9), four compounds inhibit RNase P using substrates Mh (**A**) and bipartite pre-tRNA (**B**). See Supplementary Figure S13 for a description and validation of the bipartite pre-tRNA substrate. Experiments were performed using compounds from Sigma Aldrich in standard conditions and as described in Supplementary Figures S10 and S14. The four hits are gentian violet (tris(4-(dimethylamino)phenyl)methylium chloride), juglone (5-hydroxy-1, 4-naphthoquinone)., purpurin (1,2,4-trihydroxyanthraquinone) and hematein (3,4,6a,10-tetrahydroxy-6a,7-dihydroindeno[2,1-*c*]chromen-9(6H)-one. A brief description of some properties found of these compounds is given in the Supplementary Information.

Screening was performed with the Spectrum Collection (MicroSource Discovery Systems, Inc), which comprises 2560 compounds, each dissolved in DMSO at 10 mM. For our initial screening, compounds from this collection were tested in cocktails; eight compounds were tested per well in 384-well format. The final concentration of each compound in the cocktail was 1.25 mM. For the activity assay, reactions were set up in a total volume of 20 μl (13.5 μl of activity buffer, 1.5 μl of fluorescent Mh at 3.6 μM, 3 μl of DMSO or cocktail). This set-up resulted in a final concentration of 187.5 μM for each compound. The control wells included for each experiment series were: (i) reaction with twice (3 μl) the fluorescent Mh amount to avoid a signal saturation for the rest of the wells, (ii) two reactions with 10% DMSO (no cocktail), (iii) Mh substrate only, with no ribozyme, (iv) reaction with 100 mM CaCl_2,_ which it is used as a negative control as it competes with MgCl_2_ (EDTA was not used because metal ions are essential to maintaining the structure of P RNA (41)). The reaction was initiated by adding 2 μl of holoenzyme at 333 nM and monitored as described above. Each round of experiments contained twenty different cocktails corresponding to one row of a 384-well plate (Supplementary Figures S6 and S24). Cocktails that showed inhibition (those with a slope value lower than any of the positive controls in the same plate) were verified (Supplementary Figure S7) and deconvoluted (Supplementary Figure S8) to determine the individual fragment or fragments responsible for the inhibition. At this stage, each compound from the inhibitory cocktails was tested at a final concentration of 1 mM (2 μl of the 10 mM stock). We performed a gel-based assay for the nine selected compounds to qualitatively verify the results obtained by the fluorescence assay (Supplementary Figure S9). After deconvolution, each of the final four potential positive compounds was purchased individually from a different provider (Sigma-Aldrich) and tested inhibition using the *T. maritima* Mh, pre-tRNA and bipartite pre-tRNA substrates (Figure 2 and Supplementary Figures S10–S12 and S14).

### Expression and purification of His-tagged P protein for binding assays

The gene encoding the P protein from *T. maritima* (gene synthesis performed by DNA 2.0 ATUM) was cloned into the expression vector TmP-pD454-GST as a Glutathione S-transferase (GST) fusion protein, with a TEV protease cleavage site and a His_6_-tag at the N-terminus. This vector was transformed into *E. coli* BL21(DE3)pLysS cells. Cells were then used to inoculate 3 l of Luria-Bertani media and incubated with orbital shaking at 250 rpm and 37°C until the OD_600_ reached 0.6. Protein expression was then induced with IPTG at 1 mM for 6 h. The cell pellet was collected and sonicated in 40 ml of lysis buffer (20 mM sodium phosphate monobasic, pH 8.0, 500 mM NaCl, 20 mM imidazole, 10% glycerol, 1 mM TCEP). TEV protease (700 μg) was then added directly to the lysate and incubated for 16 h at 34°C. The lysate was centrifuged at 13 000*g* and solid urea was added to the supernatant to reach a final concentration of 5 M. The sample was then purified by Ni-NTA affinity chromatography column (HisTrap FF crude, GE Healthcare) equilibrated with binding buffer (20 mM sodium phosphate monobasic, 500 mM NaCl, 5 M urea, 30 mM imidazole, pH 8.0). A 30 min wash at 5 ml/min was performed after which the protein was eluted in 20 mM sodium phosphate monobasic, 500 mM NaCl, 5 M urea, 2 M imidazole, pH 8.0. The elution was then loaded onto a gel filtration column (HiPrep 26/10 desalting, GE Healthcare) equilibrated with desalting buffer (20 mM sodium phosphate monobasic, pH 8.0, 500 mM NaCl). The eluted protein was concentrated to 5 mg/ml and stored at 19°C until needed.

### Biolayer interferometry assays (P RNA and holoenzyme)

Real-time binding assays of the holoenzyme, P RNA, P protein, the substrate minihelix and the 5′-leader to individual compounds were performed by biolayer interferometry using an Octet RED96 system (ForteBio). For the holoenzyme or P RNA, a 5′ biotinylated RNA oligonucleotide with the sequence 5′biotin-CAU**UCCAGACGAU** was immobilized on Super Streptavidin (SSA) biosensors. Then, a modified P RNA or holoenzyme was added. The P RNA modification consisted of a sequence with part of the P1 stem sequence complementary to the biotinylated oligonucleotide (AUCGUCUGGA) (Supplementary Figure S23A). P1 is far from the active site or other regions involved in specific interactions (Supplementary Figure S23B) (19). The activity of this construct was indistinguishable from the wild-type holoenzyme. Alternatively, the holoenzyme was immobilized through the His-tagged P protein from *T. maritima* at 0.05 mg/ml (3.4 μM) on Ni-NTA biosensors. Control biosensors were used to subtract non-specific binding of compounds to the biosensor surface. All assay steps (immobilization, wash, baseline, association, and dissociation) were performed in 20 mM HEPES pH 7.4, 100 mM ammonium acetate, 10 mM MgCl_2_, 2.5% DMSO. The assays were performed on black bottom 96-well microplates (Greiner Bio-One 655209) in a total volume of 200 μl, at 30°C with orbital shaking at 1000 rpm. Experiments were controlled with the software Data Acquisition 8.2 (ForteBio, Inc.). Kinetic binding parameters were calculated using Data Analysis 8.2 (ForteBio, Inc.). After subtraction of reference biosensors, the binding curves were aligned to the X- and Y-axis and the association-dissociation inter-step curve in order to get a common baseline for the association and dissociation phases.

### Biolayer interferometry assays (P protein)

Immobilization of the His-tagged P protein from *T. maritima* at 0.05 mg/ml (3.4 μM) was performed on Ni-NTA biosensors. For the initial binding tests to the P protein, a compound concentration of 1 mM was used. Periods for each step of the assay were as follows: immobilization (400 s), wash (100 s), baseline establishment (100s), association (250 s), dissociation (250 s). For each titration curve, six points were used corresponding to the 125, 62.5, 31.25, 15.6 and 7.8 μM compound concentrations. Control biosensors were used to subtract non-specific binding of compounds to the biosensor surface. All assay steps (immobilization, wash, baseline, association, and dissociation) were performed in 20 mM HEPES pH 7.4, 100 mM ammonium acetate, 10 mM MgCl_2_, 5% DMSO. The assays were performed on black bottom 96-well microplates (Greiner Bio-One 655209) in a total volume of 200 μl, at 30°C with orbital shaking at 1000 rpm. Experiments were controlled with the software Data Acquisition 8.2 (ForteBio, Inc.). Kinetic binding parameters were calculated using Data Analysis 8.2 (ForteBio, Inc.). After subtraction of reference biosensors, the binding curves were aligned to the X- and Y-axis and the association-dissociation inter-step curve in order to get a common baseline for the association and dissociation phases.

### Biolayer interferometry assays (minihelix substrate)

3′-Biotinylated minihelix was purchased from Integrated DNA Technologies (IDT) (24-mer RNA sequence, 5′-GAUCUGAAUGCGGAAACGCGCCAC-3′-Biotin). The biotinylated minihelix was immobilized on Super Streptavidin (SSA) biosensors at a concentration of 10 μM. An initial screening of compounds at 1 mM yielded a binding signal only for two compounds: gentian violet and methylene blue. For the titration experiments, 10 μM of 3′-biotinylated minihelix was immobilized for 300 s on Super Streptavidin (SSA) biosensors, followed by a wash step of 100 s and association and dissociation steps of 200 s in the presence of 6 different concentrations of compound ranging from 500 to 15.6 μM. SSA biosensors with no minihelix were used to measure non-specific binding of compounds. All other parameters (buffer, instrument control, analysis) are as described above.

### Binding of the P protein to the 5′ RNA leader and binding hindrance

A 5′ RNA leader sequence with 10-bases 5′-GGAAAAAGAU-3′ was purchased from IDT and used at a concentration of 5 μM. This sequence is the same used to obtain the crystal structure of the RNase P holoenzyme (19). The His-tagged P protein was immobilized on Ni-NTA biosensors at 0.05 mg/ml (3.4 μM). The 5′ RNA leader does not bind to the Ni-NTA biosensor surface. The steps performed on the assay and their periods were: P protein immobilization (400 s), wash (100 s), baseline (100ms) (1000 rpm), compound association (800 s), compound dissociation (200 s) (500 rpm), baseline (100 s), 5′ leader association (200 s), 5′ leader dissociation (200 s) (1000 rpm). The assay was performed at 30°C. The compounds were used at 100 μM throughout. All assay steps were performed in 20 mM HEPES pH 7.4, 100 mM ammonium acetate, 10 mM MgCl_2_, 5% DMSO.

### Molecular docking

*In silico* molecular docking of gentian violet, juglone, purpurin and hematein were performed on the RNase P protein from *Thermotoga maritima* (PDB code 1NZ0). The analysis was conducted using AutodockVina 1.2 (42). Protein and small molecule compounds’ structures were prepared by employing scripts from AutoDockTools (43). Two search boxes, with sizes 30 Å × 30 Å × 30 Å, were explored, aiming to cover the tRNA 5′-leader binding region (PDB code 3Q1R). These two boxes were centered at (*x* = 51.45, *y* = –23.71, *z* = –3.4) and (*x* = 49.01, *y* = –18.63, *z* = –8.04). When indicated, side chain flexibility was considered for F17, K51, K53 and K90. Search parameters were set to their default values. The best-suggested pose from the two searches for each ligand was selected as a starting position to perform Molecular Dynamics simulations.

### Molecular dynamics simulation

Molecular dynamics simulations were performed on the best binding modes (according to the docking score), for gentian violet, juglone and hematein. Purpurin's starting conformation corresponded to that observed in the crystal structure (PDB code: 6MAX). All systems were simulated with GROMACS 5.0.3 (44) using the AMBER99SB-ILDN all-atom force field (45) in an explicit water solvent scheme with periodic boundary conditions. The topology and force field parameters files for the gentian violet and purpurin compounds were generated with ACYPE (46). The protein protonation was performed with Molprobity (47). The water molecule model used was based on the transferable intermolecular potential with three sites (48). The solvent also included sodium chloride (NaCl) at 0.15 mM plus a small surplus of ions for electric charge neutralization. The long-range electrostatic interactions were computed using fast Particle-Mesh Ewald (PME) (49), using a grid spacing of 1.2 Å. The van der Waals and short-range electrostatic interactions were computed using a cutoff of 1.1 Å for both parameters. The neighbor list was updated every 20 steps using the Verlet cut-off scheme. All bonds were constrained using LINCS (50), enabling a simulation time step of 0.002 ps. The following startup protocol was applied to all modeled systems: Firstly, two following minimization processes with (i) 1000 steps under the steepest descendant algorithm and (ii) 500 steps using the conjugate gradient algorithm. Subsequently, the following two simulations were carried out to equilibrate the system at 300 K: (i) 500 ps in the canonical ensemble at 300 K using a V-rescale thermostat (τ = 0.1 ps) (51) and (ii) 500 ps in the isobaric-isothermal ensemble using Berendsen (52) pressure coupling at 1 bar (τ = 2.0 ps). During these processes, a restraint constant of 1000 kJ mol^−1^ nm^−2^ was applied to all heavy protein atoms and all main chain heavy atoms for the temperature and pressure equilibrations, respectively. An additional 500 ps were simulated within the isobaric–isothermal ensemble without restraints. Finally, 100 ns of molecular dynamics simulation was performed in the isobaric–isothermal ensemble using the Parrinello–Rahman barostat with no restraints (53). For all systems, the protocol was repeated three times starting from the minimized structures with a new assignment of velocities.

### Protein crystallization, fragment screening, and structure determination

P protein from *T. maritima* in 50 mM Tris–HCl pH 7.5, 0.2 mM EDTA was crystallized at 3 mg/mL in 12% PEG-1000, 100 mm sodium acetate, pH 4.8, and 200 mM potassium sulfate by vapor diffusion in a sitting drop configuration in 96-well crystallization plates (Swissci™), where 1 μl of P protein plus 1 μl of mother liquor were mixed thoroughly. Crystals appeared in 3 days at 19°C with approximate dimensions of 300 × 200 × 90 μm. Due to the frequent appearance of salt crystals of similar morphology, the presence of protein crystals was checked under a CrystaLight™ 100 UV Source (Molecular Dimensions). P protein crystals were severely damaged at concentrations of DMSO as low as 5%. Thus, purpurin was solubilized in 50% PEG-400, which proved useful in dissolving this compound entirely and maintaining the integrity of P protein crystals. From this stock, a cryoprotectant solution was prepared at a final concentration of 20 mM purpurin, which additionally contained 35% PEG-1000, 100 mM sodium acetate, pH 5.2, 20 mM potassium sulfate and 1 mM DTT. Crystals were soaked for 3 months using the solution with purpurin. Crystals were flash-frozen and stored in liquid nitrogen until needed. Diffraction data were collected at 100 K at a wavelength of 0.9785 Å at the Life Sciences Collaborative Access Team (LS-CAT) 21-ID-F and 21-ID-G beamlines at the Advanced Photon Source (Argonne National Laboratory, Argonne, IL, USA). Data were indexed and processed with XDS (54) and reduced with Aimless (55).

For fragment screening, the reference structure was solved by molecular replacement using Phaser (56), with a search model based on the structure of the P protein from *Thermotoga maritima* (PDB entry: 1NZ0). There is one monomer in the asymmetric unit, with a crystal solvent content of 40%. The presence of purpurin was initially confirmed by difference Fourier maps calculated using the structure of the wild-type enzyme.

The ligand was manually fit, and the restraints for refinement were generated using the eLBOW program of the PHENIX suite (57) using the SMILES string C1 = CC = C2C( = C1)C( = O)C3 = C(C2 = O)C( = C(C = C3O)O)O. Refinement was alternated with manual building/refinement in COOT (58), PHENIX and the PDB_REDO server (59).

The B-factor of the ligand is 42.5 versus 16.0, 25.4 and 21.1 Å^2^ for the surrounding residues Val33 Gln28 and Arg89, respectively and the density of the ligand is evident in the 2m*F*_o_ – D*F*_c_ map at a contour level of 1.5σ. σA-weighted, 2*F*_o_ – *F*_c_ simulated annealing omit maps (Supplementary Figure S19) were used to validate the presence of the ligand further.

The model presents no Ramachandran outliers. Data collection and refinement statistics are summarized in Supplementary Table S2. Figures were prepared with Pymol (The PyMOL Molecular Graphics System, Version 1.7.4, Schrödinger, LLC). The atomic coordinates and structure factors of the RNase P protein from T. maritima in the complex with purpurin have been deposited in the Protein Data Bank (PDB) with the accession code 6MAX.

## RESULTS

### A new fluorescent probe as an RNase P substrate

In this work, we designed a synthetic oligonucleotide substrate probe with a fluorophore and a non-fluorescent quencher (NFQ) at the two termini (Figure 1A, Supplementary Table S1) based on a FRET detection mechanism. If the substrate is cleaved, physical separation of the quencher from the fluorophore allows light emission that can be detected with a fluorometer (60–62). A short RNase P substrate candidate that fulfills the assay design requirements was the minihelix (Mh) pMini3pbUG (Figure 1A), whose cleavage kinetics were previously characterized (32,33) and confirmed in the present study. Substrate functionality was first tested in a polyacrylamide gel-based assay (Figure 1A) to ascertain uniform cleavage of the substrate by the RNase P holoenzyme and ensure that the appended fluorophore and quencher did not hinder catalysis. After testing the RNase P activity on the modified FRET substrate and confirming the specific cleavage (Figure 1A), we also tested the minihelix construct without the fluorescent label. The same RNase P processing pattern was observed in both cases (Figure 1A) thus supporting that the fluorophore and the quencher do not affect the ability of this substrate to be enzymatically processed by RNase P. Finally, the titration of the Mh fluorescent substrate using unlabeled pre-tRNA or Mh (Supplementary Figure S1) in solution activity assays (described below) led to a decrease in the relative fluorescence in time-course measurements.

### The Mh substrate can be used in a novel, real-time in-solution RNase P fluorescence-based assay.

The in-solution activity assays were performed at 37°C in a total volume of 20 μl on a fluorescence microplate reader in 384-well plates (Figures 1B, C, 2 and Supplementary Figures S1–S10). Supplementary Figure S2A demonstrates the stability over time and the FRET quenching efficiency of the substrate (fluorescent substrate or holoenzyme with CaCl_2_ added as a negative control). Using the Mh substrate, we did not observe RNase P activity (Supplementary Figure S2A) at 10 mM MgCl_2_. The kinetic analysis further demonstrated that this is indeed a viable and useful assay for monitoring RNase P activity (Figure 1B, left and Supplementary Figure S2B). A Michaelis–Menten fit was performed, and the kinetic parameters were calculated (Figure 1B, right). The obtained *K*_M_ and *k*_cat_ values are 1.3 μM and 6.7 min^−1^, respectively. The only previously reported values for this substrate are *K*_D_ = 2.8 μM and *k*_obs_ = 0.86 min^−1^ using the RNase P from *E. coli* (33).

### The novel fluorescence assay using Mh Is reproducible, tolerant of DMSO, sensitive to known RNase P inhibitors and amenable for high-throughput screening

**Figure 3.**
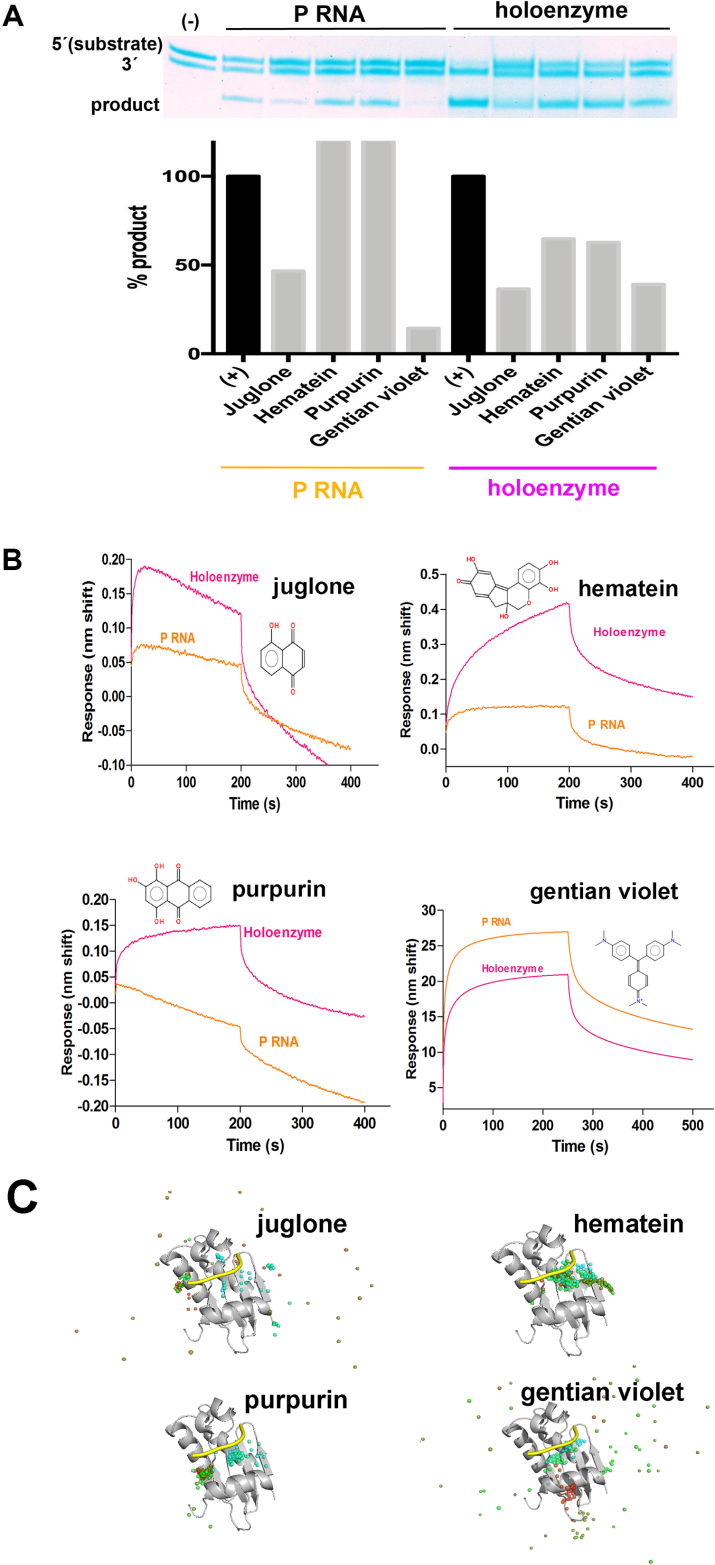
The inhibitors purpurin and hematein bind to the holoenzyme. (**A**) Top. 19% urea-PAGE demonstrates bipartite pre-tRNA substrate is processed with either P RNA or the RNase P holoenzyme from *T. maritima*. Bottom. Comparison of RNase P activity using P RNA (left) or the holoenzyme (right), indicates that purpurin and hematein do not inhibit the P RNA reaction, as derived by densitometry analysis of the lower band (product). (**B**) The relative affinities of the inhibitors for the P RNA or the holoenzyme differ. Binding sensorgrams obtained by biolayer interferometry (BLI). P RNA or RNase P holoenzyme was immobilized on super streptavidin (SSA) biosensors through a 5′-biotinylated RNA oligonucleotide complementary to a region on the P1 stem of P RNA (see Materials and Methods). Remarkably, purpurin does not bind to P RNA (orange). In contrast, gentian violet binds not only to P RNA but also to the Mh substrate (Supplementary Figure S16). (**C**) Molecular Dynamics (MD) simulations of RNase P protein in complex with the validated inhibitors. In each panel, semitransparent spheres represent the geometric center, over a 200 ns trajectory, of the corresponding tested compound. The 5′-leader (yellow) is shown for reference purposes only; it was not included in the simulation. Sphere color coding indicates the starting and final positions in blue and red, respectively. The starting complex conformation for gentian violet, juglone and hematein correspond to the best pose suggested by docking simulations. Purpurin's starting conformation corresponds to that observed in the crystal structure in complex with the P protein (PDB code: 6MAX. See Figure 5). Hematein and purpurin remain bound to the P protein throughout the dynamics protocol.

**Figure 4.**
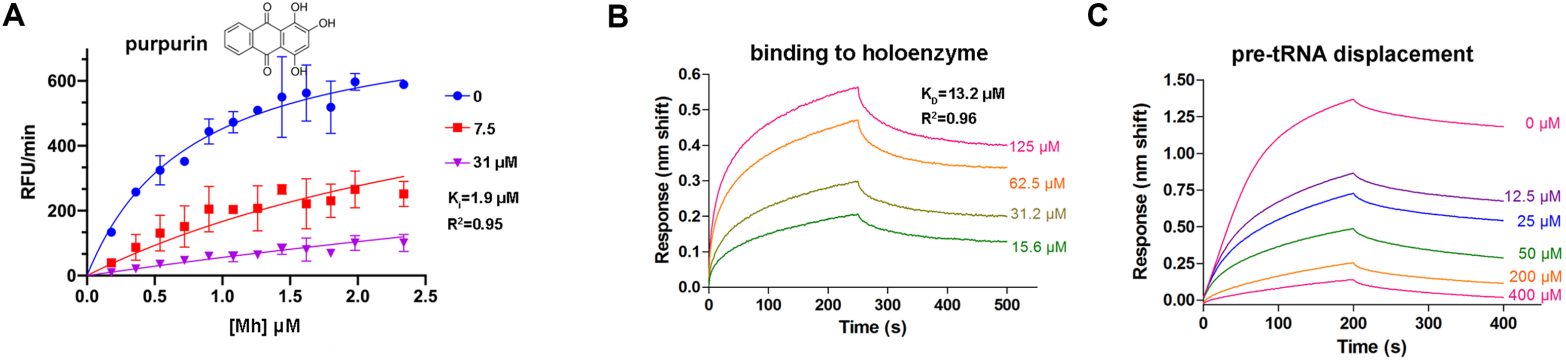
Characterization of the inhibition of *T. maritima* RNase P by purpurin. (**A**) Best global fit for inhibition of RNase P in the presence of varying concentrations of fluorescent Mh substrate. A competitive inhibition model best fit the data (*R*^2^ = 0.9549) with *K*_*i*_ = 1.9 ± 0.5 μM. Symbols represent the mean ± standard error as determined from three independent experiments at each concentration. See also Supplementary Figure S18. (**B**) The affinity of purpurin for the RNase P holoenzyme comparable to its *K*_*i*_. Binding sensorgrams obtained by biolayer interferometry (BLI). RNase P holoenzyme at 400 nM was immobilized on Ni-NTA. (**C**) Purpurin displaces bound pre-tRNA in a dose-response behavior. Binding sensorgrams obtained by biolayer interferometry (BLI). RNase P holoenzyme was immobilized on Ni-NTA biosensors, incubated with purpurin at varying concentrations (0–400 μM), and then the association and dissociation pre-tRNA was monitored. Of the four inhibitors found in this study, only purpurin exhibited a dose–response behavior in this experiment. See also Supplementary Figure S17.

Before performing this assay in a large-scale setup, we first assessed its reproducibility in 10–12 reactions usually performed in a single run (Supplementary Figure S3). To evaluate the robustness of the assay, we used a *Z*’-factor scoring method (63) for both the end-point of the assay (Supplementary Figures S3A, C) and the initial velocities of the reaction (Supplementary Figure S3B, D) (*Z*’-factor = 0.73). The linear responses in all cases were 3–5 min long (Figure 1B and Supplementary Figures S3–S10). In subsequent experiments, we considered the kinetics information given by the linear parameter to calculate the IC_50_ values for the inhibitors described in Figures 3 and 4. Most notably, we found that DMSO does not decrease the activity of RNase P on the cleavage of the Mh substrate up to a concentration of 30% (v/v) (Supplementary Figure S4). For the assays involving inhibitors (Figure 2A and Supplementary Figures S5–S10), we used a concentration of 10% DMSO.

To benchmark our assay against known inhibitors, we tested the activity of two newly-described RNase P inhibitors, methylene blue (33) (Figure 1C, left and Supplementary Figures S5A, B) and iriginol hexaacetate (26) (Figure 1C, right and Supplementary Figures S5C, D). Notably, we detected inhibition for both compounds, and IC_50_ values are in reasonable agreement with the published data (109 μM versus 62 μM (reported) for methylene blue and 29.5 μM versus 0.8 μM (reported) for iriginol hexaacetate). As expected, we did not detect inhibition by neomycin B or kanamycin B, as it is known that high Mg^2+^ concentrations nullify their effects on RNase P (64). These results corroborate the validity of the assay as we were able to detect inhibition in the same range as previously reported inhibitors and exclude non-specific interactors. Our chosen RNase P system and the developed assay (regarding substrate, real-time monitoring, volume, buffer composition, [E]/[S] ratio and DMSO tolerance) are a reliable platform to search for new inhibitors in a high-throughput manner.

### Library screening and hit validation identifies four potential inhibitors of RNase P

We performed a screen of the Spectrum Collection library (Discovery Systems, Inc.) comprising 2560 compounds (Supplementary Figure S6). A schematic view of the overall workflow and results is presented in Supplementary Figure S22. In our setup, the screening strategy involved initial testing of cocktails with eight compounds per assay/well. To select a positive hit, we considered the inhibition of the initial velocity of the reaction based on its *Z*-factor robustness (Supplementary Figures S3B and D). Several cocktails exhibited initial velocity changes that were significantly decreased (Supplementary Figure S6). In general, we further analyzed the compounds that demonstrated an initial velocity slope lower than the positive control or a negative slope (Supplementary Figure S6. Red bar corresponds to the positive control). We verified the selected cocktails by a second round of selection (Supplementary Figure S7) and finally deconvoluted to identify active compounds (Supplementary Figure S8). The potential hits were further verified at this stage by the correspondence between the fluorescence-based assay and the canonical gel-based inhibition test using lower concentrations of compounds (Supplementary Figure S9).

Using this strategy, after the initial screening (Supplementary Figure S6), 37 cocktails were selected and verified again for inhibition (Supplementary Figure S7). Of these, ten cocktails with evident inhibition were retained and then deconvoluted to deduce the 10 active compounds in each (Supplementary Figure S8). One compound, ethidium bromide, was discarded due to its known non-specific effects on nucleic acids. We thus proceeded with 9 compounds for confirmation of inhibition at a lower concentration (Supplementary Figure S9).

A gel-based inhibition assay was performed in parallel for the nine selected compounds to qualitatively verify the results obtained by the fluorescence-based assay of Supplementary Figure S9A for the nine selected compounds (Supplementary Figure S9B) (250 and 500 μM at this stage versus 1 mM for the deconvolution stage shown in Supplementary Figure S8). In this assay, the compound iodoquinol resulted in a false positive (the inhibition does not correlate in both assays, Supplementary Figures S9A and B). Pararoseaniline and emodin did not inhibit RNase P under the conditions tested in these assays (Supplementary Figure S9), nifursol is a weak inhibitor (Supplementary Figure S9), and alexidine hydrochloride seems to precipitate the holoenzyme (Supplementary Figure S9B and Supplementary Figure S8C). These results excluded five more compounds.

After this more restrictive analysis, four remaining compounds were pursued further: gentian violet (Tris(4-(dimethylamino)phenyl)methylium chloride), juglone (5-hydroxy-1, 4-naphthoquinone), purpurin (1,2,4-trihydroxyanthraquinone) and hematein (3,4,6a,10-tetrahydroxy-6a,7-dihydroindeno[2,1-*c*]chromen-9(6H)-one. We purchased these reagents from a different provider (Sigma Aldrich) and used these compounds to generate dose-response curves (Figure 2A and Supplementary Figure S10). The range of IC_50_ values was between 4.5 and 96.2 μM (Figure 2A).

### Pre-tRNA and a pre-tRNA-like substrate allow validation of the four identified hits using gel and fluorescence-based assays

First, we qualitatively validated the results obtained using the fluorescent Mh substrate by gel-based assays using a canonical pre-tRNA substrate (Supplementary Figures S11 and S12). Next, to mimic a substrate similar to the natural pre-tRNA, we designed a new probe consisting of two individual oligonucleotides (39 and 38 nucleotides for the 5′ and 3′ components, respectively) (Figure 2B, right, Supplementary Figure S13 and Supplementary Table S1). This probe, named bipartite pre-tRNA, differs from a canonical pre-tRNA in the sense that it lacks the anticodon loop and it is composed of two oligonucleotides (Supplementary Figure S13A). Binding experiments of pre-tRNA and bipartite pre-tRNA substrates to the holoenzyme using biolayer interferometry show that their affinity is comparable (*K*_D_ values of 1.8 and 3.5 nM, Supplementary Figure S13B). For the binding experiments, we tested the agreement of the affinity values obtained by BLI with previous *K*_D_ determinations of the RNase P holoenzyme:pre-tRNA and the P RNA:P protein pairs (65,40). These *K*_D_ values are in excellent agreement with the published data (Supplementary Figure S15A, B). Finally, titration of fluorescent bipartite pre-tRNA, with a similar design to fluorescent Mh, in time-course fluorescence emission experiments using unlabeled substrates shows highly similar profiles (Supplementary Figure S13C). Thus, we used fluorescent bipartite pre-tRNA as an analog that is highly similar to pre-tRNA, and we obtained dose-response curves using this substrate in the presence of the four hits obtained using fluorescent Mh (Figure 2B and Supplementary Figure S14). As the sensitivity to DMSO for this assay using the bipartite pre-tRNA substrate was very high (no more than 2% DMSO was tolerated, similar to (26,17), we used 10% PEG 200 instead.

Overall, the fluorescent screening strategy—using both a model substrate, tolerant to high DMSO concentrations and a pre-tRNA-like substrate, useful to demonstrate the inhibition properties under low monovalent and Mg^2+^ conditions—demonstrates its utility to find new small molecule inhibitors directed against RNase P.

### Purpurin and hematein bind to the RNase P holoenzyme

Gel-based assays performed using the bipartite pre-tRNA substrate (Figure 3A) under a single buffer condition where both P RNA and the holoenzyme display activity demonstrated that the hits purpurin and hematein inhibit the reaction catalyzed by the RNase P holoenzyme but not the P RNA alone (Figure 3A). The gel-assay using the bipartite pre-tRNA substrate allowed us to distinguish this behavior clearly. In agreement with this result, binding assays with immobilized P RNA or holoenzyme using a 5′ biotinylated RNA oligonucleotide (Figure 3B) show that purpurin does not bind to P RNA and hematein binds only slightly to P RNA (Figure 3B). In contrast with the behavior of these two hits, gentian violet inhibits the reaction *via* P RNA (Figure 3A) and also binds more strongly to it (Figure 3B). Moreover, unlike the other three hits, gentian violet binds to the biotinylated minihelix substrate in the micromolar range (Supplementary Figure S16). The hit juglone displayed a less clear behavior in these assays (Figure 3A, B).

**Figure 5.**
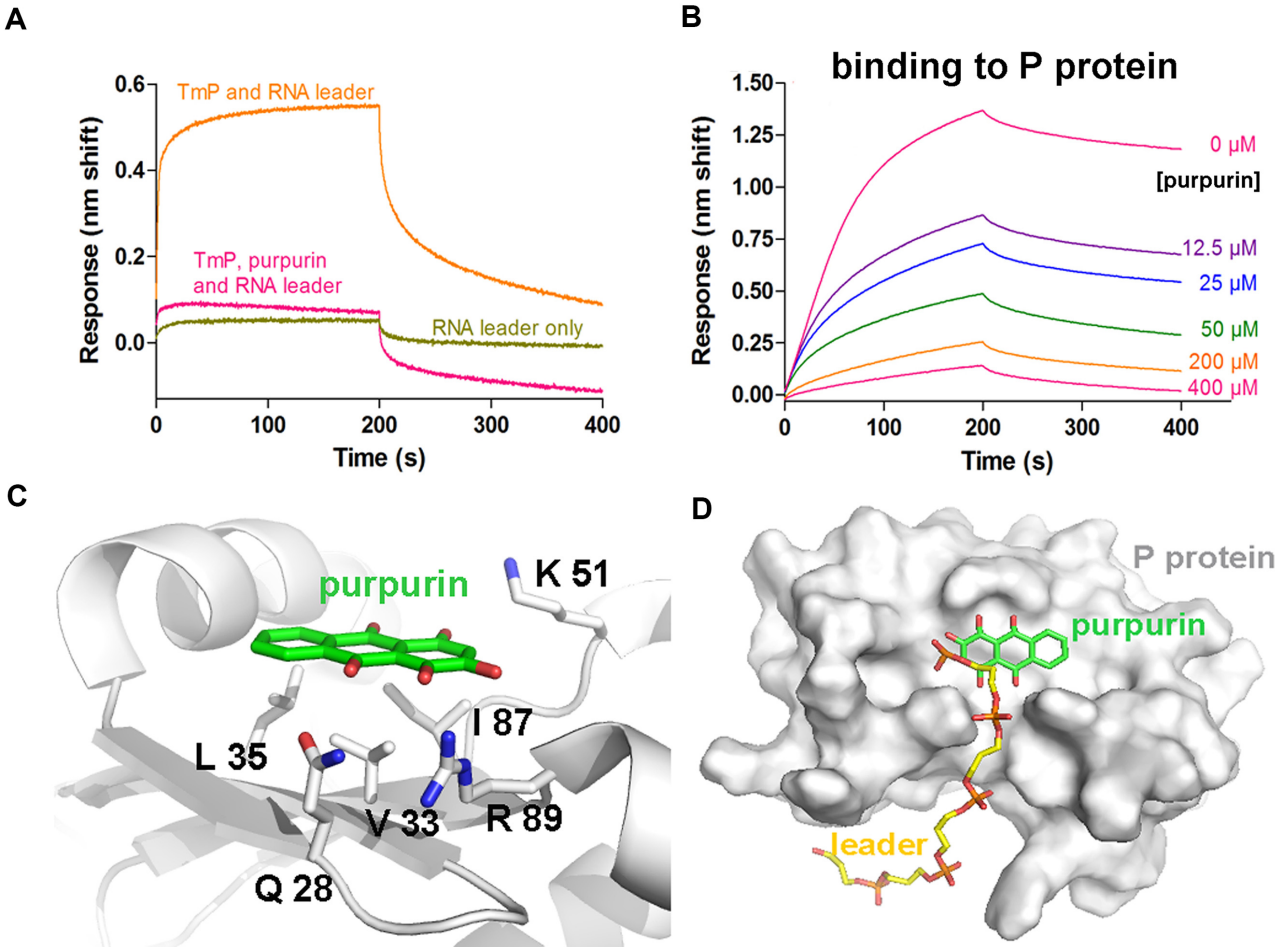
Purpurin binds the P protein. (**A**) Purpurin hinders the binding of the P protein to the 5′ RNA leader. Association and dissociation of the 5′ RNA leader to the P protein (TmP) is measured over time. P protein was immobilized on Ni-NTA biosensors while purpurin was assayed at 100 μM and the 5′ RNA leader at 5 μM. A 10-nt leader was used for these experiments. See Supplementary Figure S10 for the experimental design and testing of the other three inhibitors. (**B**) Purpurin binds to the P protein from *T. maritima* in a dose-response manner (*K*_D_ = 6.8 μM). Binding sensorgrams obtained by BLI. P protein was immobilized on Ni-NTA biosensors after which its association and dissociation from indicated compounds were monitored. Sensorgrams were fit to a 1:1 Langmuir binding model. (**C**) Location of purpurin (green) on the *T. maritima* P protein as determined by X-ray crystallography (PDB code: 6MAX; see Supplementary Figure S19 and Supplementary Table S2). (**D**) Location of the 5′ RNA leader (yellow) on the P protein in the context of the RNase P holoenzyme in complex with tRNA (19) (PDB code: 3Q1R). In both cases, residue Arg 89 (labeled in panel C) forms interactions with the different ligands.

Given that the main difference between P RNA and the holoenzyme is the presence of the P protein, we performed a preliminary interrogation of potential inhibitor interactions between the P protein and each of the four hits using docking and Molecular Dynamics (MD) (Figure 3C and Supplementary Figure S18). Small molecule docking studies of gentian violet, juglone and hematein were performed on the model obtained from crystallographic data of the RNase P protein from *T. maritima* (PDB codes 1NZ0, 3Q1R). Next, Molecular Dynamics (MD) simulations were performed on the four compounds with the best-scoring docking poses or the purpurin position as observed in complex with the P protein by X-ray crystallography (Figure 5C, PDB code: 6MAX). While the dynamics preserve the overall protein structure, the binding modes of the tested compounds were affected (Figure 3C and Supplementary Figure S20). While hematein and purpurin rearranged themselves into a new binding mode around the initial docking or crystallographic pose around the P protein (Figure 3C and Supplementary Figure S20), juglone and gentian violet did not adopt a stable arrangement (Figure 3C and Supplementary Figure S20).

Overall, the activity, binding, docking and MD experiments point to gentian violet as a non-specific inhibitor that binds to RNA, juglone as an inhibitor with an ambiguous mechanism and hematein and purpurin as inhibitors of *T. maritima* RNase P holoenzyme which may specifically target the P protein.

### Purpurin inhibits RNase P holoenzyme through a competitive mechanism and shows a dose-response relationship on its binding to the holoenzyme and pre-tRNA displacement

We tested whether any of the four validated hits were capable of displacing the canonical substrate pre-tRNA from the holoenzyme in a binding assay. Purpurin displays a dose-response relationship and remarkably, none of the other three hits demonstrated this behavior (Figure 4C, Supplementary Figure S17). Together, all of the data collected for purpurin are in agreement with the activity and binding criteria to validate an RNase P inhibitor (Table 1). Further characterization shows that the *K_i_* value of purpurin (Figure 4A, Supplementary Figure S18), obtained from an adjustment to a competitive inhibition model, is in general agreement with the *K*_D_ value obtained for RNase P holoenzyme-purpurin (Figure 4B, Table 1).

**Table 1.** Summary of inhibition and affinity parameters for the four identified hits

Compound name	Mh IC_50_ (μM)	Bipartite pre-tRNA IC_50_ (μM)	Targets RNA	Dose–response displacement of pre-tRNA	*K_i_* and *K*_d_ (holoenzyme) (μM)
Gentian violet	7.3 ± 1.6	16.2 ± 2.1	++ (substrate)	No	ND
Juglone	12.8 ± 1.9	11.9 ± 1.7	+	No	ND
Purpurin	96.2 ± 21.7	13.1 ± 2.3	-	Yes	1.9 ± 0.4, 13.2 ± 2.6
Hematein	4.5 ± 0.9	87.9 ± 19.7	-	No	ND

### Purpurin has a binding site on the P protein in a region important for substrate binding

To further explore the possible binding of purpurin to the P protein, we tested the binding interference of an RNA oligonucleotide with the sequence of a 5′leader in the presence of this compound (Figure 5A). Remarkably, purpurin interferes with the binding of the leader to the P protein (Figure 5A). The three other hits were also tested in this manner (Supplementary Figure S19). Juglone and gentian violet do not impede the leader binding (Supplementary Figure S19). We discounted hematein because, although it displayed leader-binding hindrance (Supplementary Figure S19), unlike purpurin, it lacked a dose-response relationship on pre-tRNA displacement in the presence of the holoenzyme (see Figure 4C for purpurin). We next determined that purpurin binds to the P protein with micromolar affinity in a dose-response relationship (Figure 5B). Finally, the crystal structure of *T. maritima* P protein in complex with purpurin (Figure 5C, DSupplementary Figure S21 and Table S2) shows a binding region of the compound located in a hydrophobic patch formed by residues Val 33, Leu 35 and Ile 87. Additional hydrogen-bonding interactions are observed between purpurin and Gln 28 and Arg 89. Arg 89 is a conserved protein residue, essential for efficient *T. maritima* RNase P activity, that potentially contacts the pre-tRNA leader binding region (Figure 5D) (38). Taken together, the binding and X-ray crystallography results indicate the existence of a region in the P protein that is sensitive to the binding of inhibitory compounds of *T. maritima* RNase P and that this region is also crucial for substrate binding.

## DISCUSSION

In this work, we developed and implemented novel methods to discover and validate inhibitory compounds against bacterial RNase P and gain insight into the structure and function of this ribozyme. Although FRET assays have been used for high-throughput screening of RNA-based targets (66,61,62), this is the first description of this type of method tailored for RNase P. The most noteworthy differences between the proposed method compared to the recent advances described by (26) are the DMSO tolerance (provided by the use of the Mh substrate), the salt tolerance (which reduces non-specific binding) and the development of a novel bipartite pre-tRNA substrate (which allows hit validation). In particular, the DMSO tolerance of the new assay allows for additional chemical space to be probed beyond what was previously explored. It should be noted however that, given the assay design, inhibitors that alter the fidelity of processing might appear as false negatives in the fluorescence-screening step. It should also be considered that there might be a quenching effect from cleaved 5′ leader that could lead to an underestimation of the catalytic activity. Despite these potential issues, the presented method offers a powerful way to discover new inhibitors under a variety of conditions and substrates.

Additionally, this work presents the first binding screening method amenable for high-throughput for RNase P. The method may be useful for enhanced characterization of known inhibitors (26) or to demonstrate specific binding to the holoenzyme, P RNA or P protein (67,68). Notably, this method provided insights into the inhibitory mode of action via monitoring the pre-tRNA or the leader binding hindrance to the holoenzyme or the P protein. Overall, the results obtained point to the P protein as a promising target for the yet undiscovered inhibitors.

We also used molecular docking, molecular dynamics, and X-ray crystallography to elucidate the possible site of action for the validated hits. P protein is an ideal target for these techniques. Due to its small size (117 aa, 14 kDa), efficient MD protocols are executed successfully. The protein crystallizes in 3 days, the crystals diffract to high resolution (∼1.5 Å), a structure of the holoenzyme is known, and the critical binding sites have been identified (19). Most importantly, the crystallization precipitant (PEG 1000) is fully compatible with the solvent used to solubilize fragments (PEG 400 50%). However, it has been challenging to obtain complexes of the P protein with different ligands, probably due to steric hindrance in the crystal packing. Nonetheless, we succeeded in obtaining the crystal structure of the P protein in complex with purpurin, which binds in a region important for the 5′-leader interaction with the protein (38). This region includes R89, a highly conserved residue that makes putative contacts with the phosphate backbone of N-5 of the pre-tRNA leader. The mutation of this residue has substantial effects on RNase P activity (38). These results point to the P protein as an attractive component for targeted inhibition.

Table 1 shows a summary of the four validated hits and some parameters analyzed in this work. Most importantly, purpurin is the only compound that shows similar IC_50_ values using the fluorescent substrates Mh and bipartite pre-tRNA and presented a dose-response relationship on the pre-tRNA displacement assay. Moreover, the obtained *K*_D_ value to the holoenzyme and the *K*_*i*_ are in reasonable agreement. Holistically, this work demonstrates the necessity to consider several corroborating approaches before committing to an initial small-molecule inhibitor hit. While purpurin binds somewhat weakly, its relatively low molecular weight (256 Da), may allow its further optimization.

Three of the found hits—juglone, hematein, and purpurin—have characteristics of known non-specific inhibitors in drug screens, specifically catechol and quinone groups that are known to undergo addition reactions, complex with divalent metal ions, or act as redox cyclers (69–71). On the other hand, several lines of evidence point to purpurin as a potential inhibitor amenable to further optimization. This compound maintains inhibition even at remarkably high concentrations of MgCl_2_ (100 mM) and ammonium acetate (400 mM), shows a dose–response relationship in binding the holoenzyme, binding P protein and displacing pre-tRNA, and it presents a specific binding site on the P protein, as revealed by the co-crystal structure.

Taken together, this work offers the following technological advances that may be more widely applied to the study of RNA-protein complexes: (i) establishment of biolayer interferometry as a real-time technique for measuring ligand interactions with all the components of RNase P (P RNA, P protein and substrate); (ii) demonstrating the utility of a novel, pre-tRNA-like substrate, which may be useful for the study of other tRNA-processing enzymes and (iii) highlighting the use of low molecular PEGs as substitutes for DMSO in the study of small ligands in complex with macromolecules. This work offers a multi-technique platform for inhibitor discovery and indicates, for the first time, that the P protein is a promising target for future antibiotics.

## DATA AVAILABILITY

Atomic coordinates and structure factors for the reported crystal structure have been deposited in the Protein Data Bank under accession number 6MAX.

## Supplementary Material

gkz285_Supplemental_File
