# Supplementary material for: A screening platform to monitor RNA processing and protein-RNA interactions in ribonuclease P uncovers a small molecule inhibitor
Source: Nucleic Acids Res. 2019 Apr 18;47(12):6425–38. doi: 10.1093/nar/gkz285 (PMC6614837; doi:10.1093/nar/gkz285)
Supplement: gkz285_Supplemental_File [file gkz285_supplemental_file.pdf]

Supporting Information

Supplementary Figures

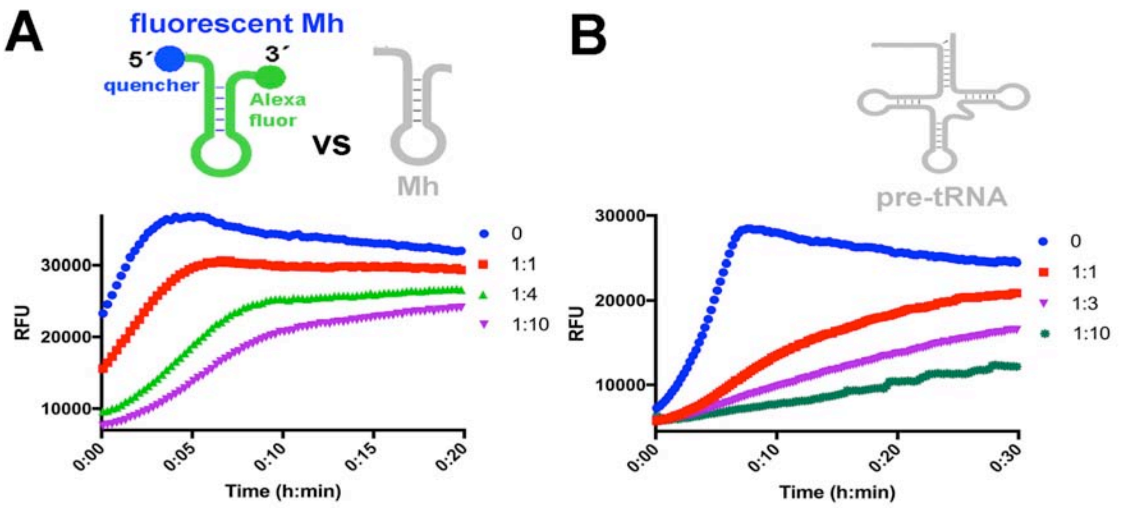

Supplementary Figure 1. The fluorescent minihelix (Mh) substrate is cleaved by bacterial RNase P. Titration of unlabeled Mh (A), or pre-tRNA (B) led to a decrease in the relative fluorescence in time-course measurements (see Figure 1B and Supplementary Figure 2). The molar ratios of (labeled: unlabeled) probe used are indicated to the right of each graph.

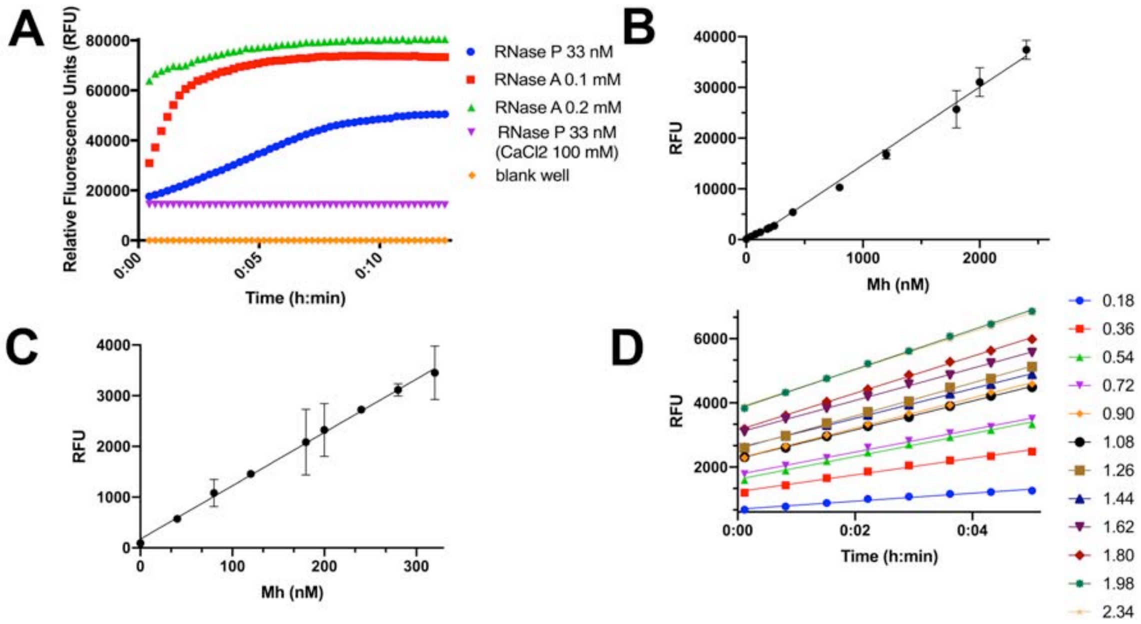

Supplementary Figure 2. Kinetic assays of RNase P with the fluorescent substrate show a typical Michaelis-Menten enzymatic behavior. (**A**) Fluorescence emission time course at 37°C. Ribonuclease A (RNase A) was used as a positive control and to estimate the maximum fluorescence given by the complete cleavage of the Mh substrate (panels B and C). These controls also demonstrate the specific minihelix (Mh) substrate fluorescence in the reaction assays upon cleavage and the fluorescence stability over time. Note that the signal of the Mh only sample overlaps with the holoenzyme-$CaCl_2$ sample and is not displayed for clarity. (**B** and **C**) Standard curves using RNase A (2 μl of the enzyme at 100 μg/ml in a total reaction volume of 20 μl in buffer TH 100 mM, 400 mM ammonium acetate, 100 mM $MgCl_2$). These curves (R^2=0.9891 and 0.9506) for B and C, respectively) allowed for the conversion of Relative Fluorescent Units (RFU) to substrate concentration. Thus, for example, 572 RFU correspond to 40 nM of the substrate. (**D**) Representative plots of the linear region (initial velocities) of kinetic assays are shown. RNase P holoenzyme was incubated at 11 nM, with increasing Mh concentrations as indicated. The calculated slope and the substrate concentration were used to determine the velocity in nM substrate/min (Figure 1B).

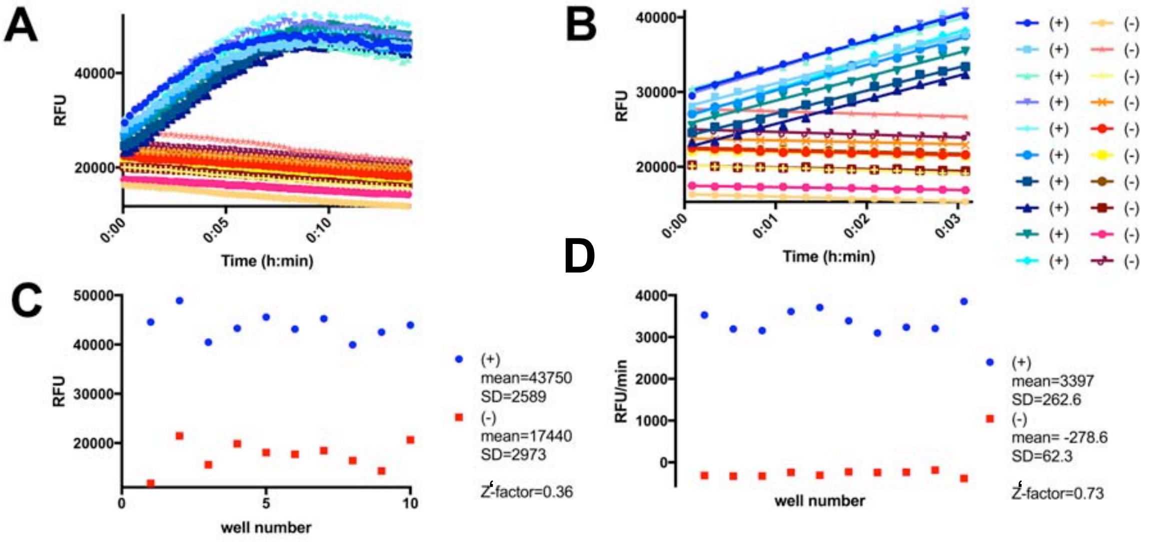

Supplementary Figure 3. FRET assays of RNase P with the fluorescent substrate are reproducible. (**A**) Representative plots of kinetic assays performed in a single run. The standard assay (SA), (+), was performed at 37°C in a total volume of 20 µL and contained RNase P holoenzyme at 33 nM, substrate minihelix (Mh) at 270 nM, 10% DMSO and activity buffer (AB). The negative control assay (-) contained 100 mM $CaCl_2$ in place of $MgCl_2$. Ten to twelve wells were monitored simultaneously in a typical assay. (**B**) Zoomed view of the linear region of the kinetic assays shown in panel A. Fluorescence readings were performed every 15-20 seconds. Linear regression calculations were done using the GraphPad Prism 7 software. (**C**) The coefficient Z'-factor (1) was used as an indicator of reproducibility and was calculated based on the relative fluorescence units (RFU) in the plateau phase (taken at 20 minutes). The Z'-factor has a value of 0.36, indicating that the assay is not usable unless performed in duplicate. All the assays presented in this work were either measured in triplicate or confirmed in subsequent assays. (**D**) The calculated Z'-factor is 0.73 for the slope values determined from panel B, indicating that the assay is robust. The IC_{50} values reported in Figures 1C and 2 were calculated according to this kinetic parameter. There is a negative drift in fluorescence in the case of the negative control. However, it should be noted that the mean value of it (-279) is much smaller than the signal of the positive control (3397). We monitored and verified this ratio in all the subsequent performed assays.

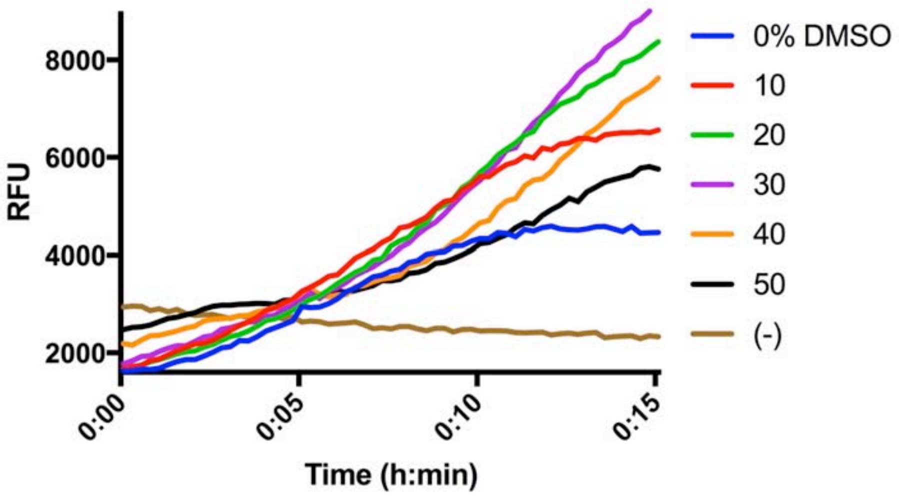

Supplementary Figure 4. RNase P cleaves the fluorescent substrate in the presence of the solvent dimethyl sulfoxide (DMSO). Representative plots of kinetic assays performed in standard conditions (SA, Figure 3) in the presence of different concentrations of DMSO. The activity is stimulated up to a concentration of 30% (v/v) DMSO and decreases abruptly at higher concentrations. The assays depicted in Figures 1C, 2A and Supplementary Figures 5-10 were measured in the presence of 10% DMSO in standard conditions (SA), as defined in Supplementary Figure 3.

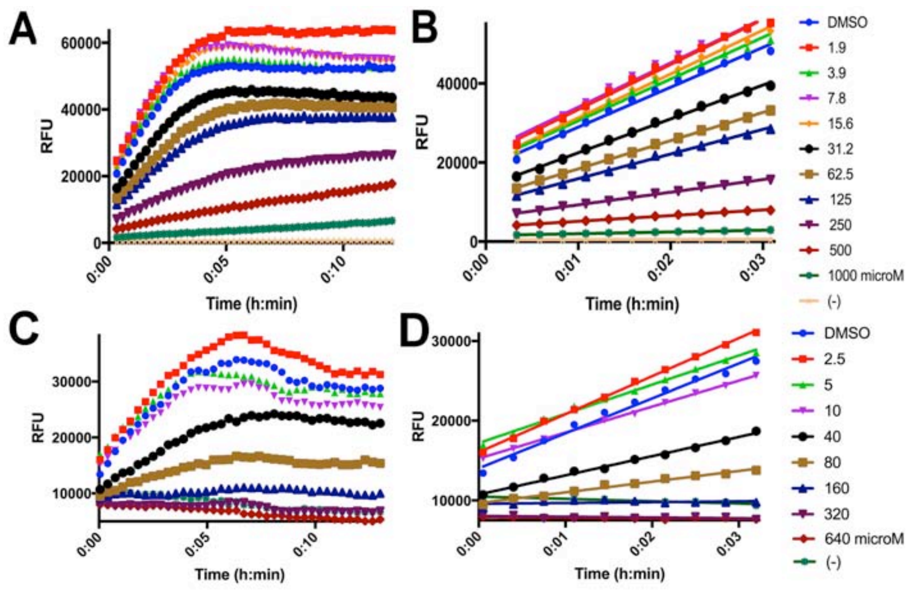

Supplementary Figure 5. The reported RNase P inhibitors methylene blue and iriginol hexaacetate inhibit bacterial RNase P from *Thermotoga maritima*. (A-B). The inhibition of RNase P by methylene blue. (**A**) Representative plots of kinetic assays performed in the presence of the different concentrations of methylene blue (indicated right). The DMSO label corresponds to the activity in the presence of 10% of DMSO. (**B**) The linear portions of the kinetic assays shown in panel A. The slope values were obtained by linear regression. **(C-D). Inhibition of RNase P by iriginol hexaacetate.** (**C**) Representative plots of kinetic assays performed in the presence of the different concentrations of iriginol hexaacetate. (**D**) Linear portion of the kinetic assays shown in panel C.

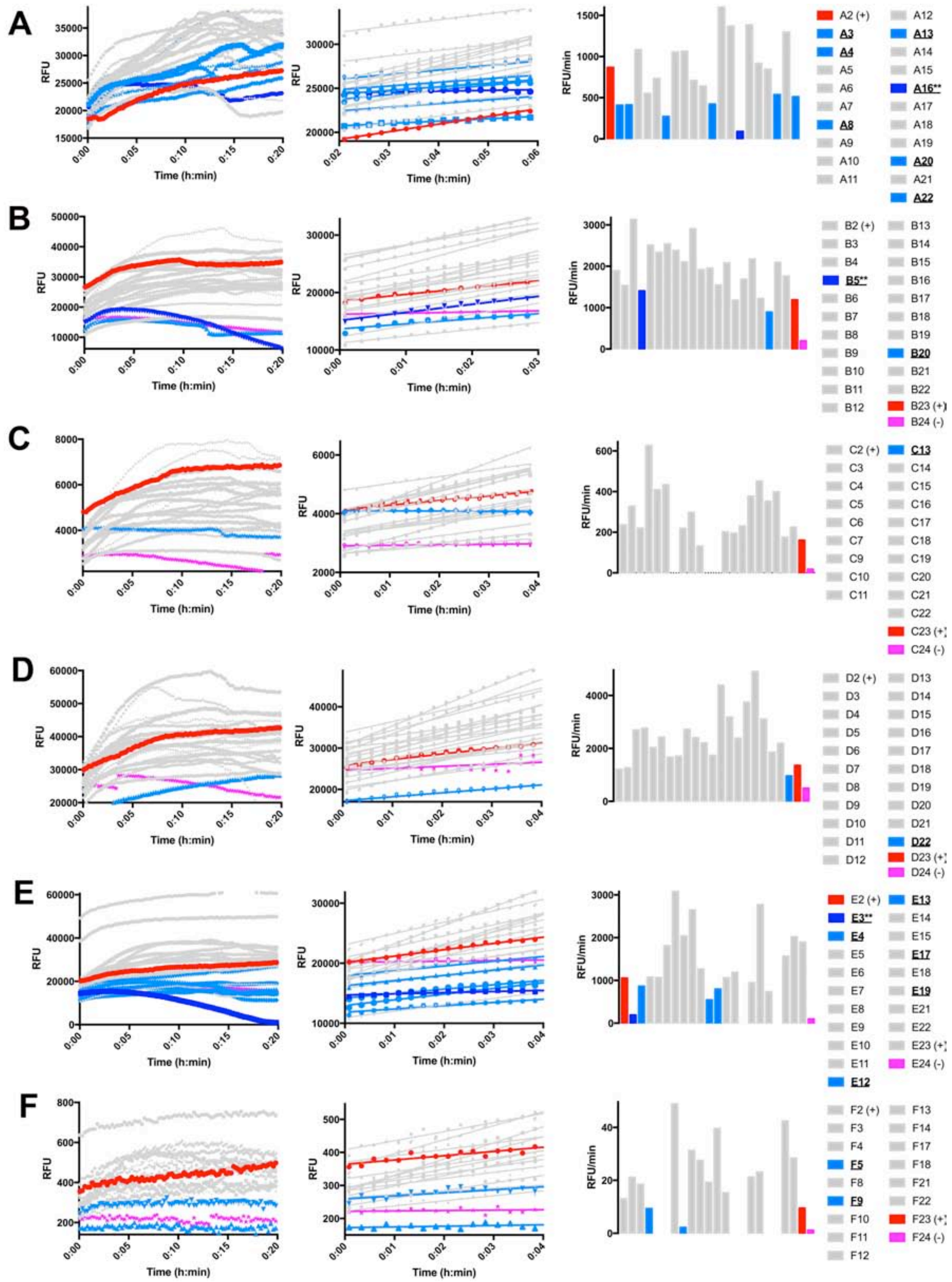

Supplementary Figure 6. See legend below panel P.

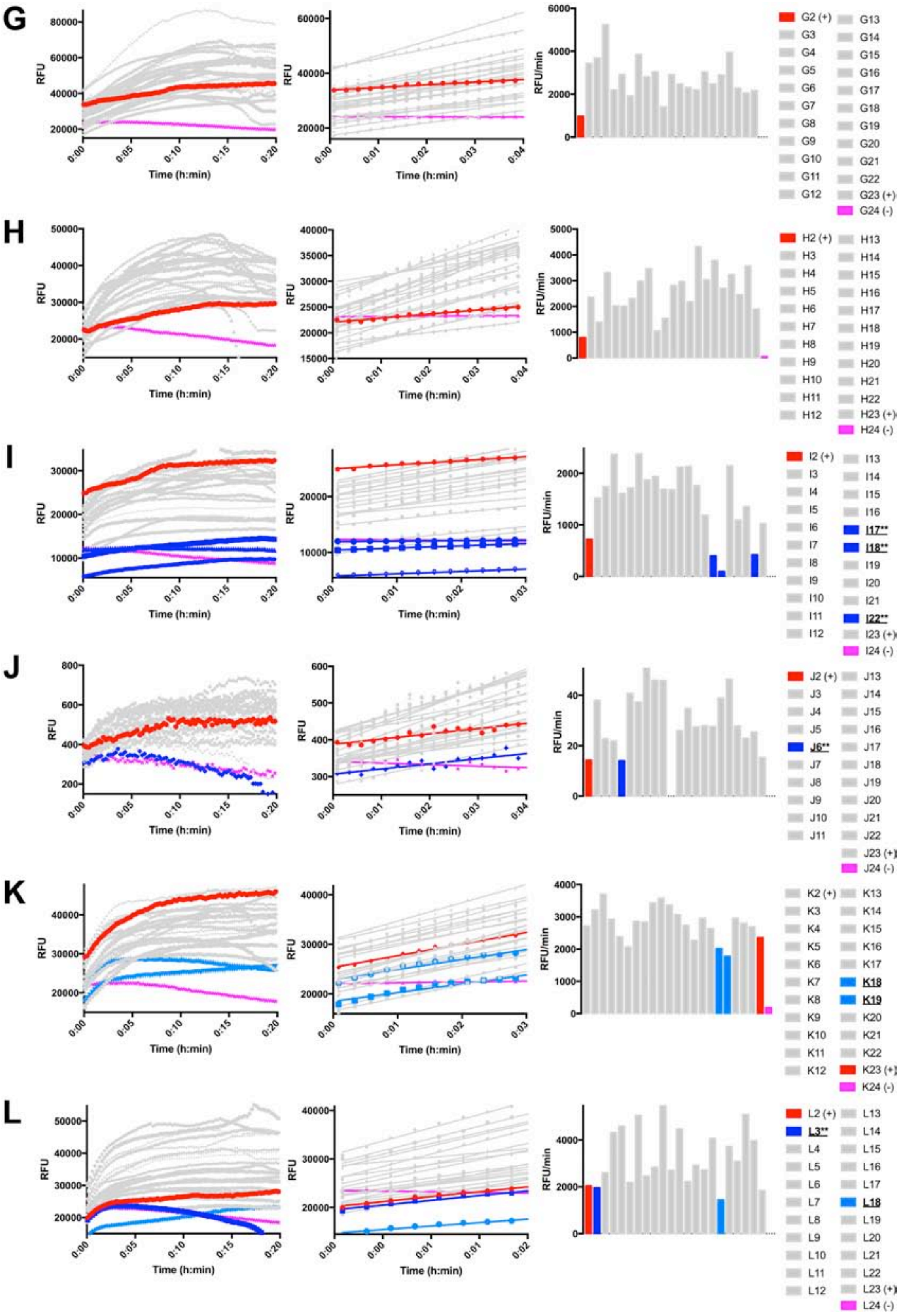

Supplementary Figure 6. See legend below panel P.

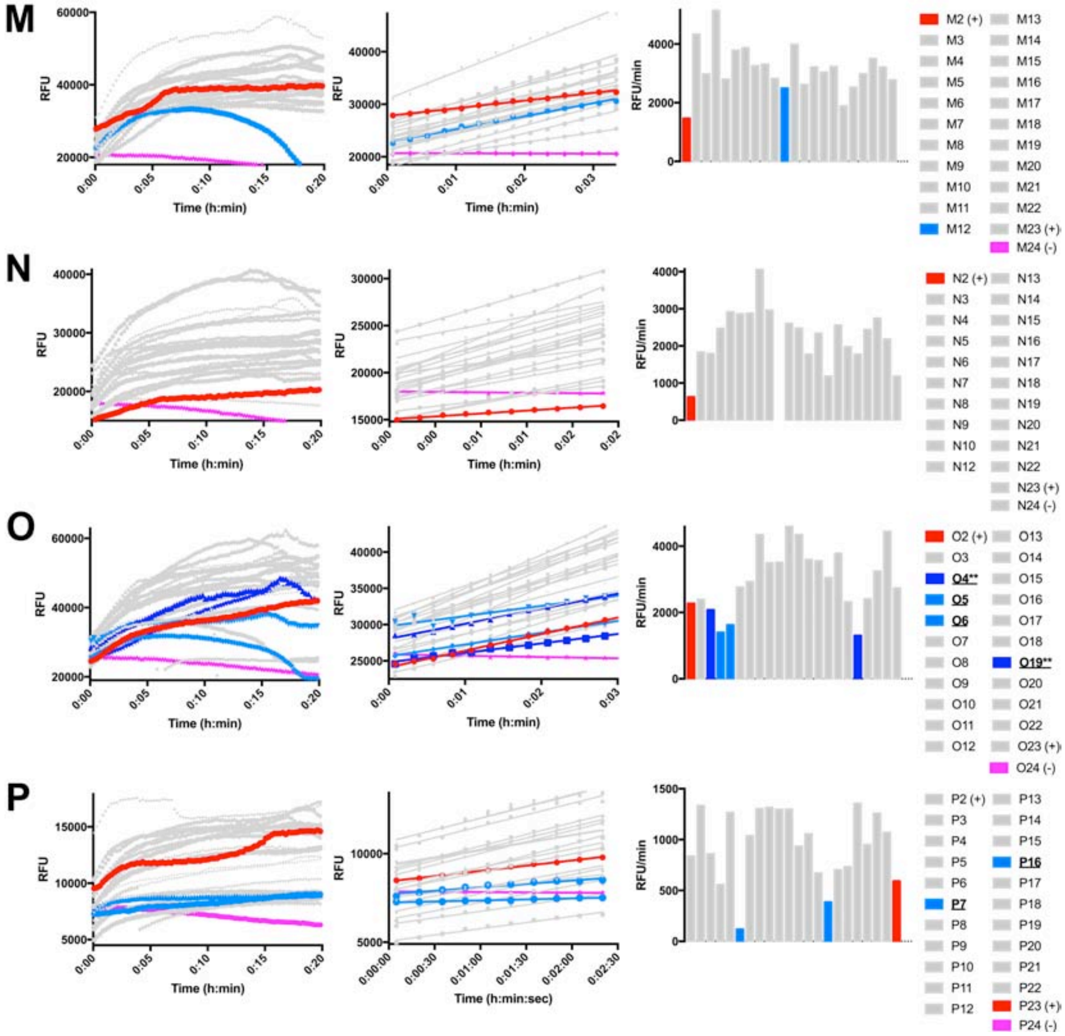

Supplementary Figure 6. Library screening in solution is suitable using the fluorescent substrate. 2560 compounds from the Spectrum Collection (MicroSource Discovery Systems, Inc), available as 10 mM solutions in DMSO in 1536-well plates, were dispensed into 384-well microplates and then grouped into cocktails of 8 members (each member of the cocktail had a resulting concentration of 1.25 mM). Two cocktails were formulated per plate column, as each plate has 16 wells per column. The inhibition assay was done in a total volume of 20 μL in black 384-well microplates, as described in the Methods section. At this screening stage, 21 cocktails (an almost full row of a 384 plate) were tested per round. See Supplementary Figure 24 for an illustration of the experimental setup. (**Left**) Plots of the kinetic assays performed in the presence of the different cocktails. (**Center**) The linear region of the kinetic RNase P assays. (**Right**) Slope values obtained from the linear regression performed on the different experiments shown in the center panels and plotted as bar graphs. The cocktails selected for verification are marked in blue, and the cocktails that tested positively after verification (Supplementary Figure 7) are marked in dark blue and with a double asterisk.

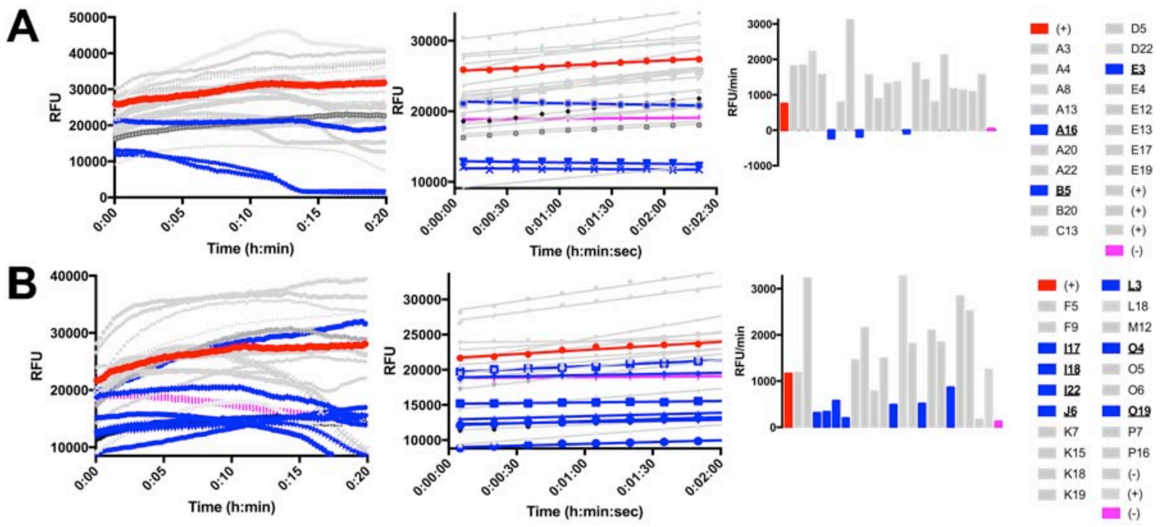

Supplementary Figure 7. Cocktail hit verification. The assay conditions were identical to those used for the library screening (Supplementary Figure 6). The cocktails that were deconvoluted (Supplementary Figure 8) are marked in blue.

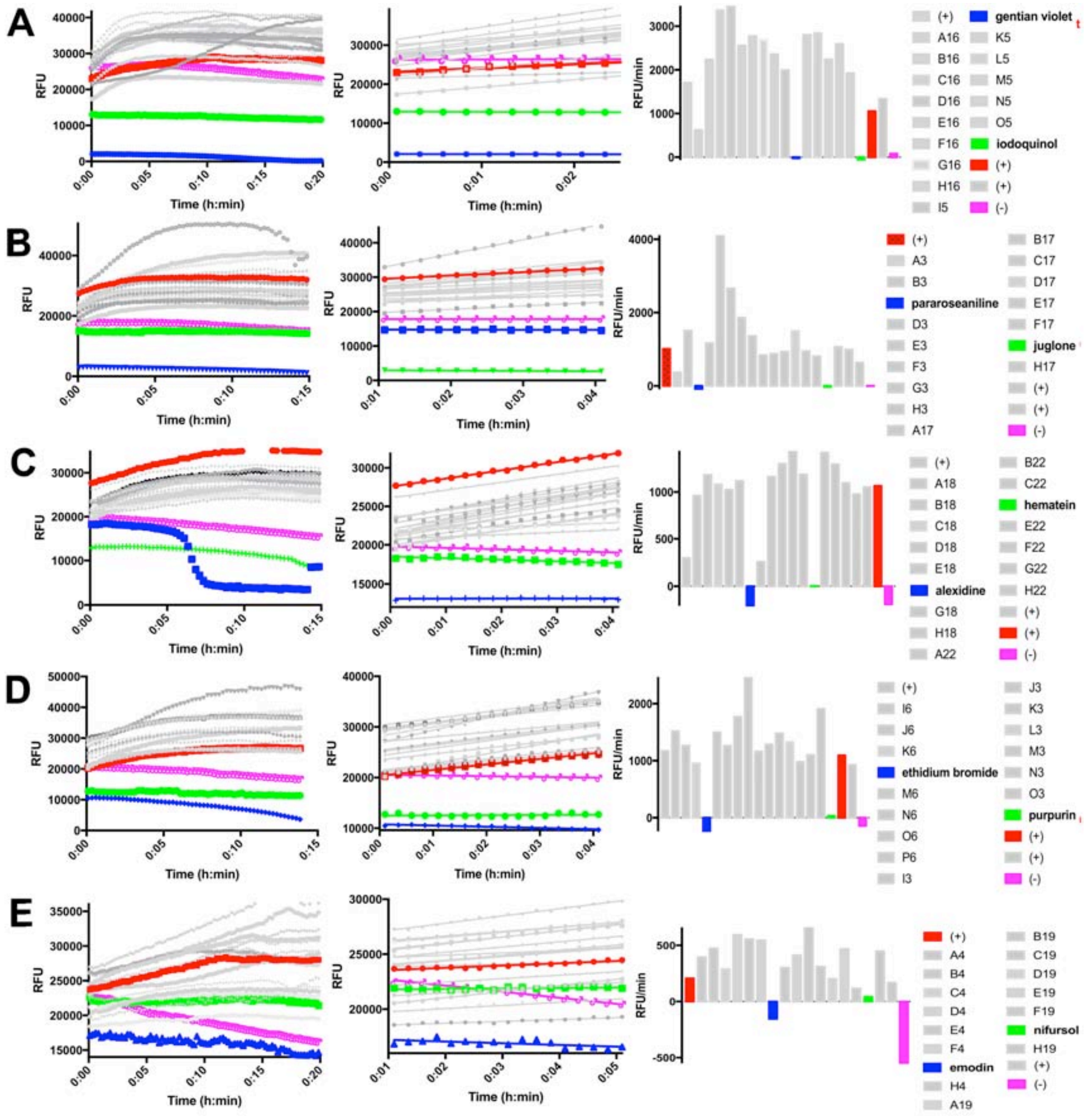

Supplementary Figure 8. Hit deconvolution of the 2560-compound library screening. (**Left**) Plots of the kinetic assays performed in the presence of compounds from validated cocktails (Supplementary Figure 7). The concentration of each compound in these assays was of 1 mM. (**Center**) Plot of the initial velocities (kinetics of product formation) taken from the experiment shown in the left panel. (**Right**) The slope values were obtained from the linear regression performed on the different experiments shown in the center panels and plotted as bar graphs. Each of the cocktails, except A16, tested positively for one or two (cocktail B5) compounds. In order to be highly restrictive for this pilot study, only the compounds with a slope value close to or below zero were considered as positive hits. Ethidium bromide was not considered further in subsequent assays (Supplementary Figure 9).

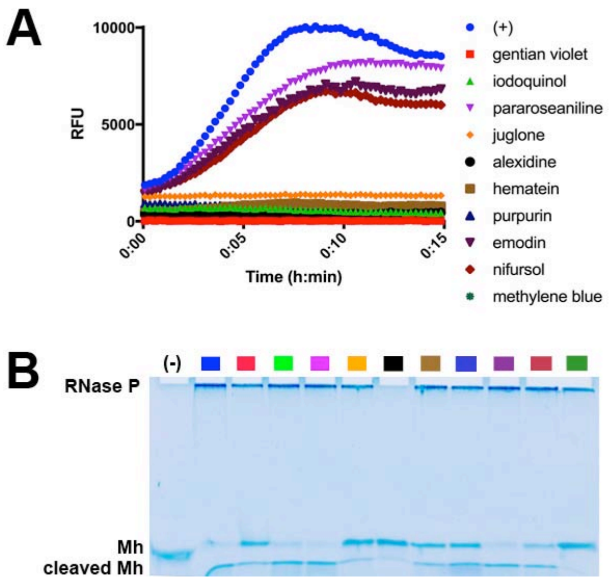

Supplementary Figure 9. Consistent results between fluorescence- and gel-based assays. (**A**) Plots of fluorescent kinetic assays performed in standard conditions in the presence of the different hits at a concentration of 250 μM (four-fold lower concentration as compared to Supplementary Figure 8). (**B**) End-point assays of the RNase P holoenzyme from *Thermotoga maritima* in the presence of the non-fluorescent minihelix substrate and the different inhibitor hits at 500 μM, as assessed by 20% urea-PAGE. The holoenzyme at 0.5 μM was preincubated at 37°C for 30 minutes in the presence of the individual compounds, centrifuged for 15 minutes at 12000g and incubated with the Mh substrate at 18 μM at 37°C for 15 minutes. At this stage, iodoquinol was the only compound whose behavior was not corroborated in both assays (panels A and B). Pararoseaniline and emodin did not inhibit RNase P under the conditions tested in the experiments reported in this Figure. Nifursol is a weak inhibitor (panel B). Alexidine hydrochloride precipitates the holoenzyme (RNase P was not observed in the well corresponding to the black square) (See also panel C in Supplementary Figure 8). The final, validated, positive hits were gentian violet (red), juglone (orange), hematein (brown) and purpurin (blue).

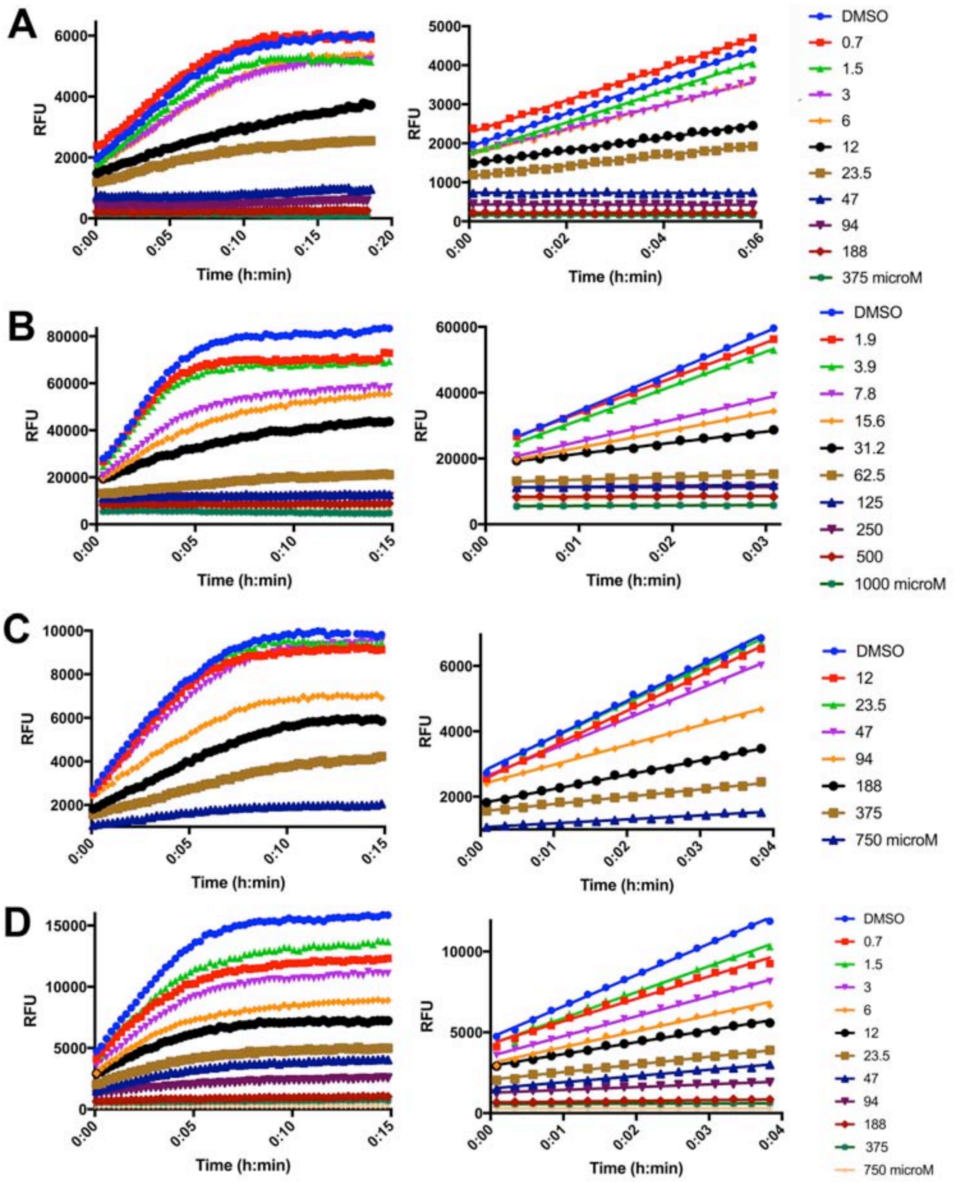

Supplementary Figure 10. Four validated hits inhibit RNase P holoenzyme from *Thermotoga maritima* in the micromolar range using the substrate Mh. Experiments were performed using compounds from Sigma Aldrich in standard conditions and as described in Supplementary Figure 5. (**A**) Gentian violet (Tris(4-(dimethylamino)phenyl)methylium chloride) (R^2=0.9577). (**B**) Juglone (5-hydroxy-1, 4-naphthoquinone) (R^2=0.9366). (**C**) Purpurin (1,2,4-Trihydroxyanthraquinone) (R^2=0.9254). (**D**) Hematein (3,4,6a,10-Tetrahydroxy-6a,7-dihydroindeno[2,1-c]chromen-9(6H)-one (R^2=0.8921). A brief description of some properties found of these compounds is given at the end of this Supplementary Information. The R^2 value indicating the fitting to the dose-response curves shown in Figure 2A is indicated in parenthesis.

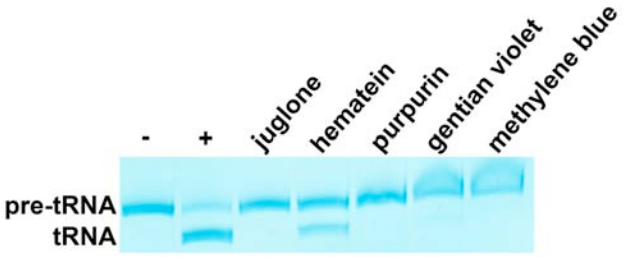

Supplementary Figure 11. End-point urea-PAGE RNase P activity assays using a canonical pre-tRNA substrate are in agreement with the fluorescence inhibition experiments. RNase P holoenzyme from *T. maritima* is inhibited by the four validated hits, as observed in an 18% urea-PAGE end-point assay. RNase P holoenzyme was incubated in the presence of 10% DMSO (+) or different concentrations of compound for 30 min at 37°C. See also Supplementary Figure 12.

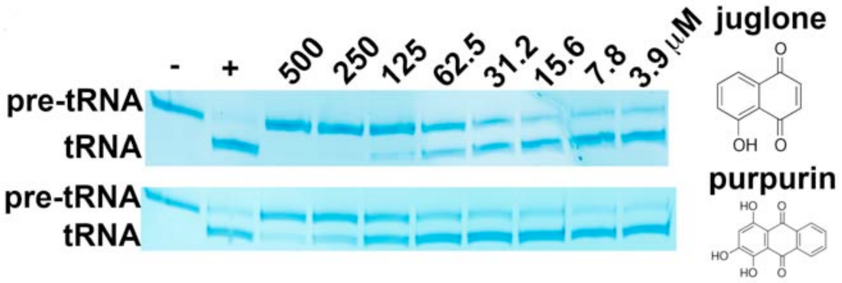

Supplementary Figure 12. End-point urea-PAGE RNase P activity assays using a canonical pre-tRNA substrate are in agreement with the fluorescence inhibition experiments using the minihelix substrate. Compound-dependent disappearance of the pre-tRNA substrate, with a simultaneous accumulation of the tRNA product, confirms the inhibition in the context of a biological substrate for the inhibitors (juglone and purpurin). The assays were performed under the following conditions: 66 nM RNase P holoenzyme, in a total volume of 20 μL in activity buffer (33 mM Tris, 66 mM HEPES pH 7.4, 400 mM ammonium acetate, 100 mM $MgCl_2$) were incubated in the presence of 10% DMSO (+) or different concentrations of compound for 30 min at 37°C. Then, pre-tRNA was added to a final concentration of 1.6 μM and the reaction was incubated at 55°C for 25 min before gel loading.

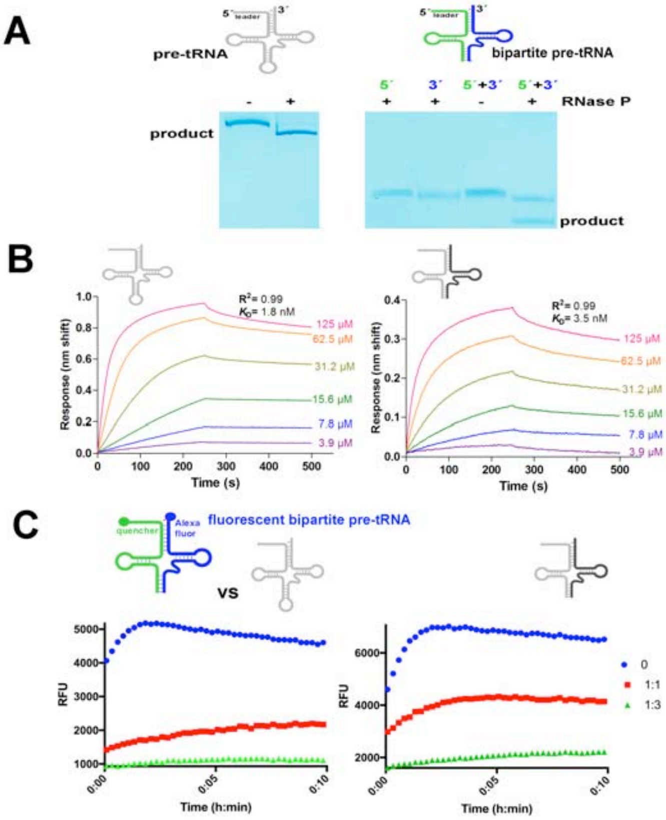

Supplementary Figure 13. A new fluorescent (bipartite pre-tRNA) substrate is highly similar to the canonical substrate pre-tRNA. (**A**) Top. Schematic representation of the canonical 84 nucleotides pre-tRNA (left) and a 77-nucleotide (right) bipartite pre-tRNA substrate that excludes the anticodon loop sequence and is composed of two oligonucleotides. Bottom. RNase P holoenzyme from *Thermotoga maritima* cleaves the bipartite pre-tRNA substrate, as observed by 19% urea-PAGE. (**B**) The affinity of pre-tRNA or bipartite pre-tRNA for the holoenzyme is very similar. Binding sensorgrams obtained by biolayer interferometry (BLI). RNase P holoenzyme at 400 nM was immobilized on Ni-NTA biosensors via a His-tagged P protein for 300 s, followed by wash and baseline steps of 100 s each and the association and dissociation steps of 250 s. Sensorgrams were fitted to a 1:1 Langmuir binding model, with excellent adjustments reflected by the high R^2 values obtained. (**C**) Titration of unlabeled pre-tRNA (left) or bipartite pre-tRNA (right) using a fluorescent bipartite pre-tRNA substrate ensures that the Relative Fluorescence decreases in time-course fluorescence emission experiments. The molar ratios of labeled: unlabeled probe indicated to the right of each graph.

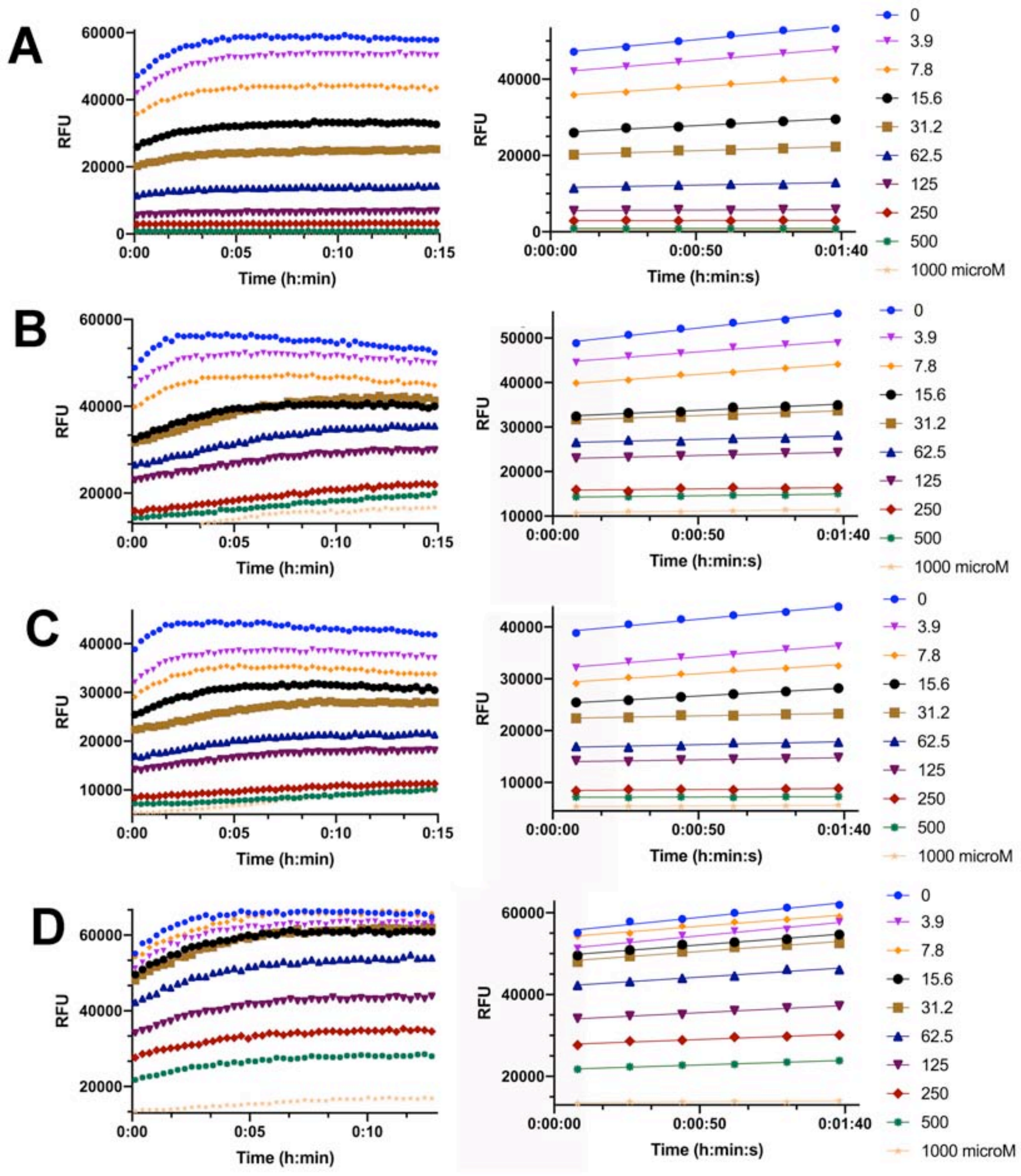

Supplementary Figure 14. Four hits inhibit RNase P holoenzyme from *Thermotoga maritima* in the micromolar range using the substrate bipartite pre-tRNA. (**A**) Gentian violet (R^2=0.9648). (**B**) Juglone (R^2=0.9479). (**C**) Purpurin (R^2=0.9469). (**D**) Hematein (R^2=0.8976). The zero "0" label corresponds to the plot of the activity using 33 nM RNase P in the presence of 10% of PEG 200 (in place of DMSO) at 20°C in a reaction buffer consisting of 50 mM ammonium acetate, 5 mM $MgCl_2$, 100 mM Tris pH 8.0. The R^2 value indicating the fit to the dose-response curves shown in Figure 2A is indicated in parenthesis.

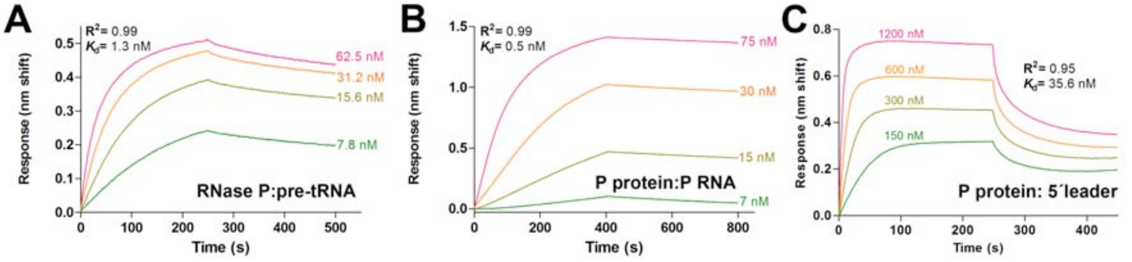

Supplementary Figure 15. The K_D values obtained from binding assays using biolayer interferometry (BLI) are in agreement with those previously reported. Each panel depicts binding sensorgrams obtained by biolayer interferometry (BLI) for the indicated interactions. (**A**) RNase P holoenzyme:pre-tRNA. Reconstituted RNase P holoenzyme from *T. maritima* at 800 nM was immobilized through His-tagged P protein on Ni-NTA biosensors for 600 s in 33 mM Tris, 66.6 mM HEPES, 200 mM ammonium acetate, 10 mM $MgCl_2$, pH 7.4, followed by wash and baseline steps of 100 s each. The pre-tRNA in buffer HAAMD (20 mM HEPES pH 7.4, 100 mM ammonium acetate, 10 mM $MgCl_2$, 5% DMSO or 5% PEG 200) was measured against RNase P in concentrations ranging from 62.5 to 7.8 nM in a two-fold dilution series. Sensorgrams were fit globally to a Langmuir 1:1 binding model. The calculated K_D value of 1.3 nM is in agreement with the reported values of 0.2-0.4 nM for the *E. coli* and *B. subtilis* holoenzymes (2); (**B**) P protein:P RNA. His-tagged P protein was immobilized on Ni-NTA biosensors at 3.5 μM (0.05 mg/mL) for 300 s in buffer 33 mM Tris, 66.6 mM HEPES, 200 mM ammonium acetate, 10 mM $MgCl_2$, pH 7.4, followed by wash and baseline steps of 100 s each. Binding of P RNA from *T. maritima* was measured at concentrations from 75 nM to 7 nM in a two-fold serial dilution manner. The calculated K_D value of 0.5 nM is in agreement with the reported value of 0.4 nM for the P protein from *E. coli* (3) (**C**) P protein:5′-leader. P protein at 3.5 μM was immobilized in Ni-NTA biosensors for 300 s, followed by wash and baseline steps of 100 s each and the association and dissociation steps of 250 s with different concentrations of a 10 nucleotide 5′ leader. The buffer used during all the steps was 20 mM HEPES pH 7.4, 100 mM ammonium acetate, 10 mM $MgCl_2$, 5% DMSO. The calculated K_D is 35.6 nM. Sensorgrams were fit to a simple 1:1 binding model. Data from figures 9-11 to were analyzed with the Data Analysis 8.2 version (ForteBio, Inc).

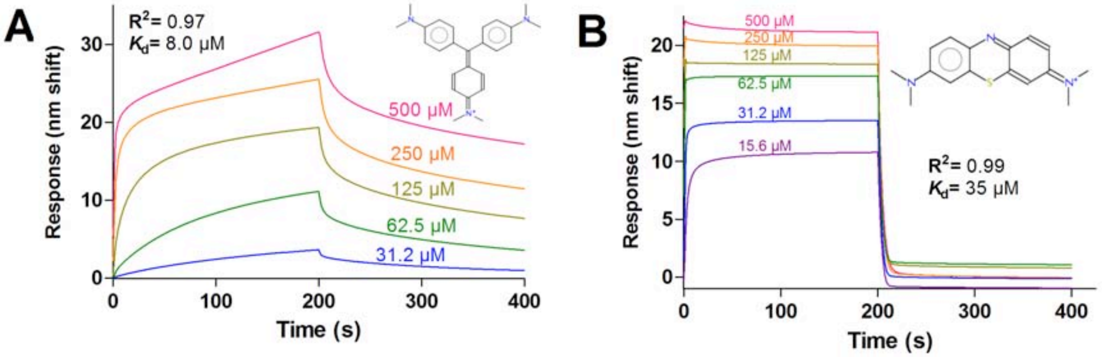

Supplementary Figure 16. Binding assays monitor the interaction of compounds with the minihelix (Mh). Gentian violet and methylene blue bind in a dose-dependent relationship to the artificial minihelix substrate. Depicted in the figure are the sensorgrams demonstrating the interaction between the minihelix and **(A)** gentian violet (K_D=8 µM) and **(B)** methylene blue (K_D=35 µM). A 3′-biotinylated substrate minihelix at 10 µM was immobilized on super streptavidin (SSA) biosensors for 300 s.

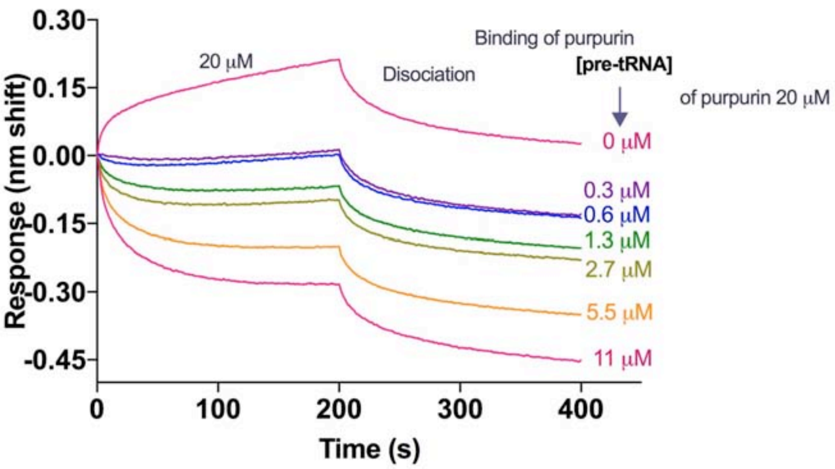

Supplementary Figure 17. pre-tRNA displaces bound purpurin in a dose-response behavior. This experiment represents the opposite configuration to the one shown in Figure 4C, indicating a true competition between purpurin and pre-tRNA binding sites.

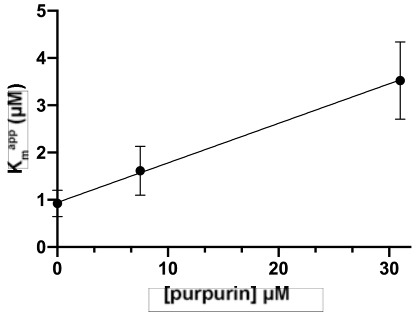

Supplementary Figure 18. Purpurin is a competitive inhibitor. Inhibition of RNase P in the presence of varying concentrations of fluorescent Mh substrate. Linear plot (R^2=0.9038) of the apparent K_m obtained from the experiments shown in Figure 4A as a function of inhibitor concentration, illustrating that the inhibitor range used is well suited for the experimentally determined K_i value.

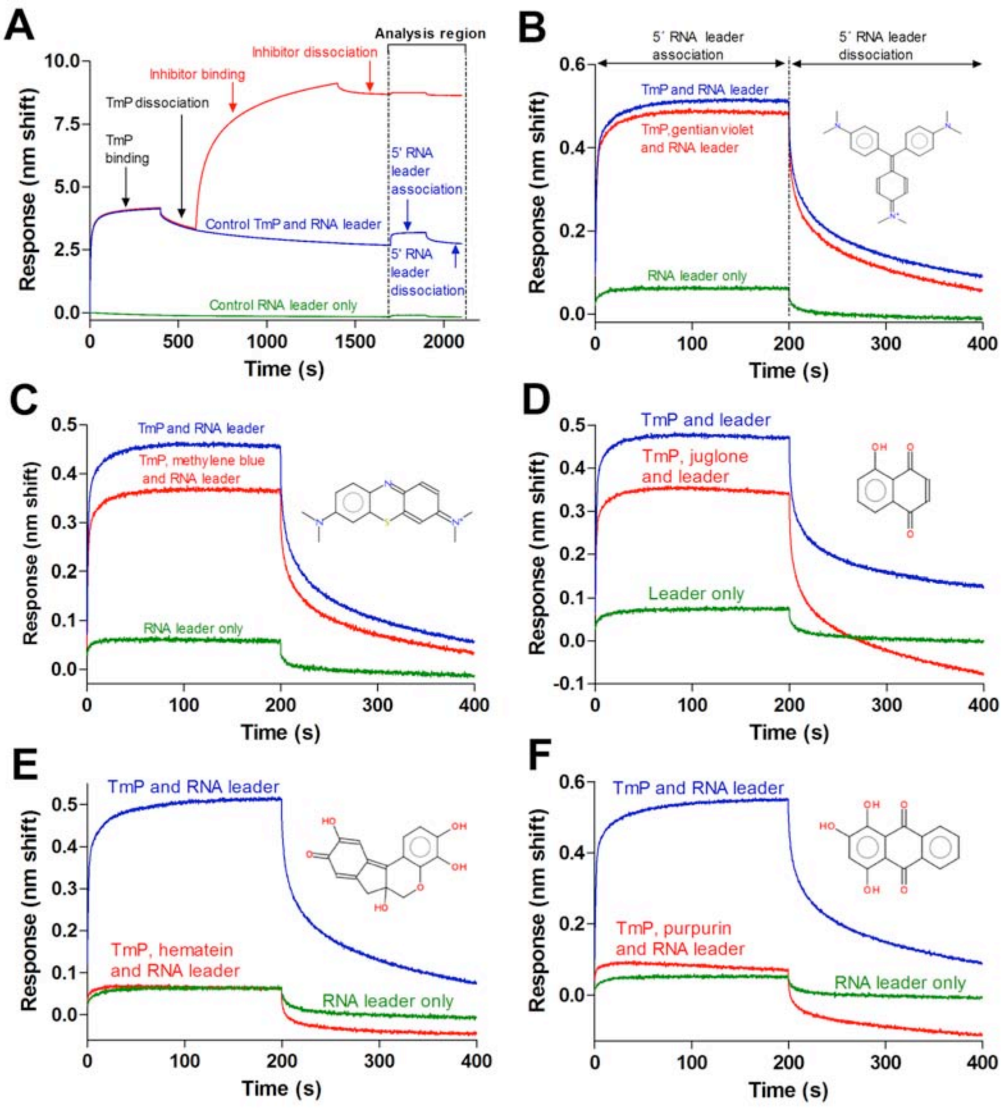

Supplementary Figure 19. Hematein and purpurin hinder the binding of the P protein to the 5′ RNA leader. (A) Experimental design of 5′ RNA leader binding hindrance experiments**.** Figures B-F are based on the analysis region depicted in panel A, where association and dissociation of the 5′ RNA leader to the P protein (TmP) is measured over time. P protein at 3.5 μM was immobilized on Ni-NTA biosensors for 300 s, while compounds were assayed at 100 μM and the 5′ RNA leader at 5 μM. Gentian violet (**B**) or methylene blue (**C**) did not interfere with RNA leader binding, in contrast with hematein **(E)** and purpurin **(F)**.

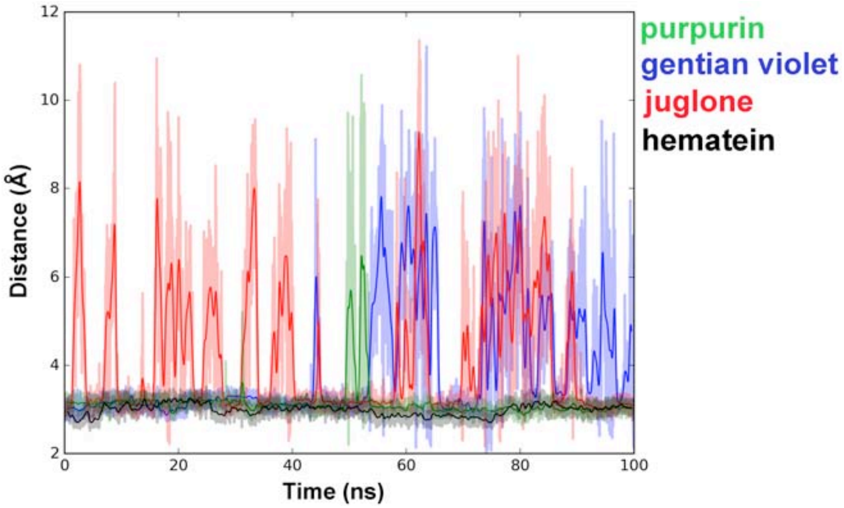

Supplementary Figure 20. Molecular docking and molecular dynamics simulations of the suggested complexes between P protein and the inhibition hits show that hematein and purpurin bind to the P protein. Average curves for the minimal distance between the P protein and the associated compounds. For each system (P protein in complex with a corresponding inhibitory compound), the average curves (shaded region), with a 50-frame window moving average (solid line), were computed from three independent simulation runs. The RMSD was computed employing the suggested docking structure as a reference and considering the Cα and heavy atoms (non-hydrogens) for P protein and the associated compound, respectively. The distance was computed considering the closest distance between the heavy atoms of the P protein and the associated compound.

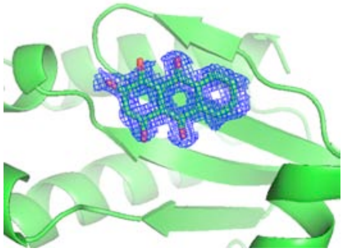

Supplementary Figure 21. Crystal structure of P protein from *T. maritima* in complex with purpurin. 2mFo-DFc, simulated annealing electron density map (blue mesh) contoured at 1σ around purpurin complexed to the P protein. Purpurin dissolved in 50% PEG 400 was soaked at 20 mM for 3 months.

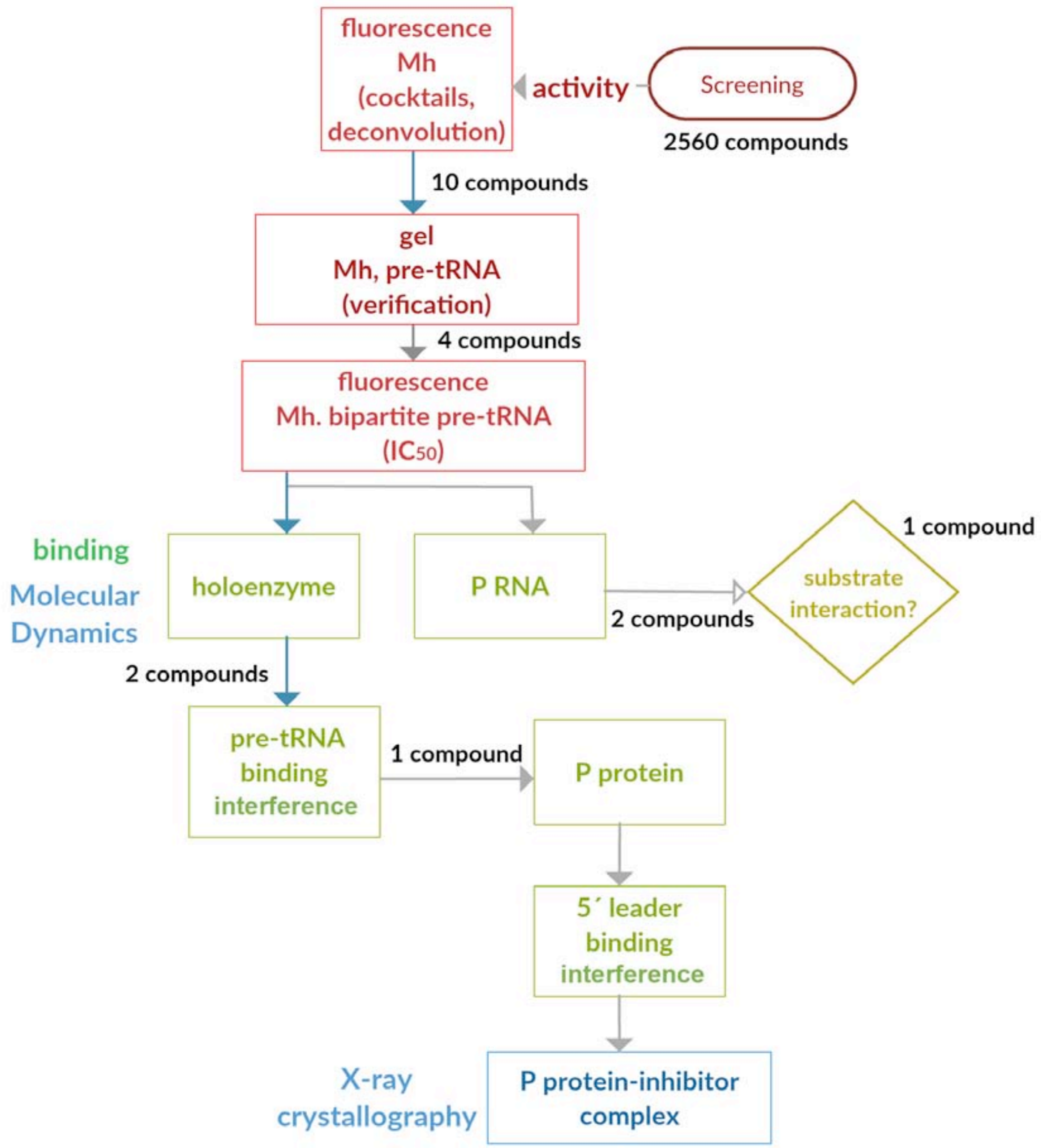

Supplementary Figure 22. Schematic view of the overall activity-binding-structure workflow used in this work to discover novel bacterial RNase P inhibitors. Red indicates studies involving activity (by fluorescence or in-gel). Green indicates binding studies by biolayer interferometry (BLI). Blue indicates structural studies using the P protein by docking, molecular dynamics, and X-ray crystallography.

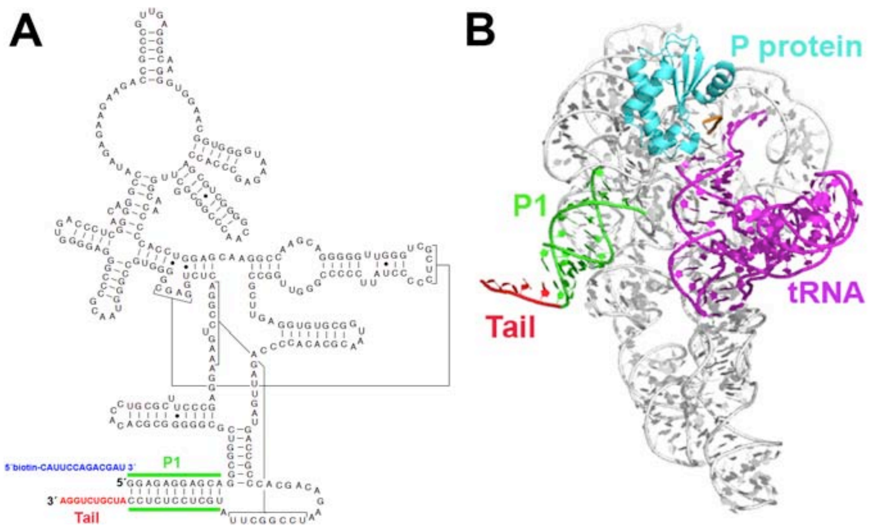

Supplementary Figure 23. Diagram showing the design of the P RNA sequence to allow its capture by a biotinylated RNA oligo for BLI studies. (**A**) Modified P RNA sequence with part of the P1 stem complementary to a biotinylated RNA oligonucleotide (AUCGUCUGGA), which in turn may bind to streptavidin-coated biosensors. (**B**) Location of the P1 modification (in red, marked as tail) of P RNA on the three-dimensional structure of the holoenzyme in complex with tRNA (PDB code 3Q1R).

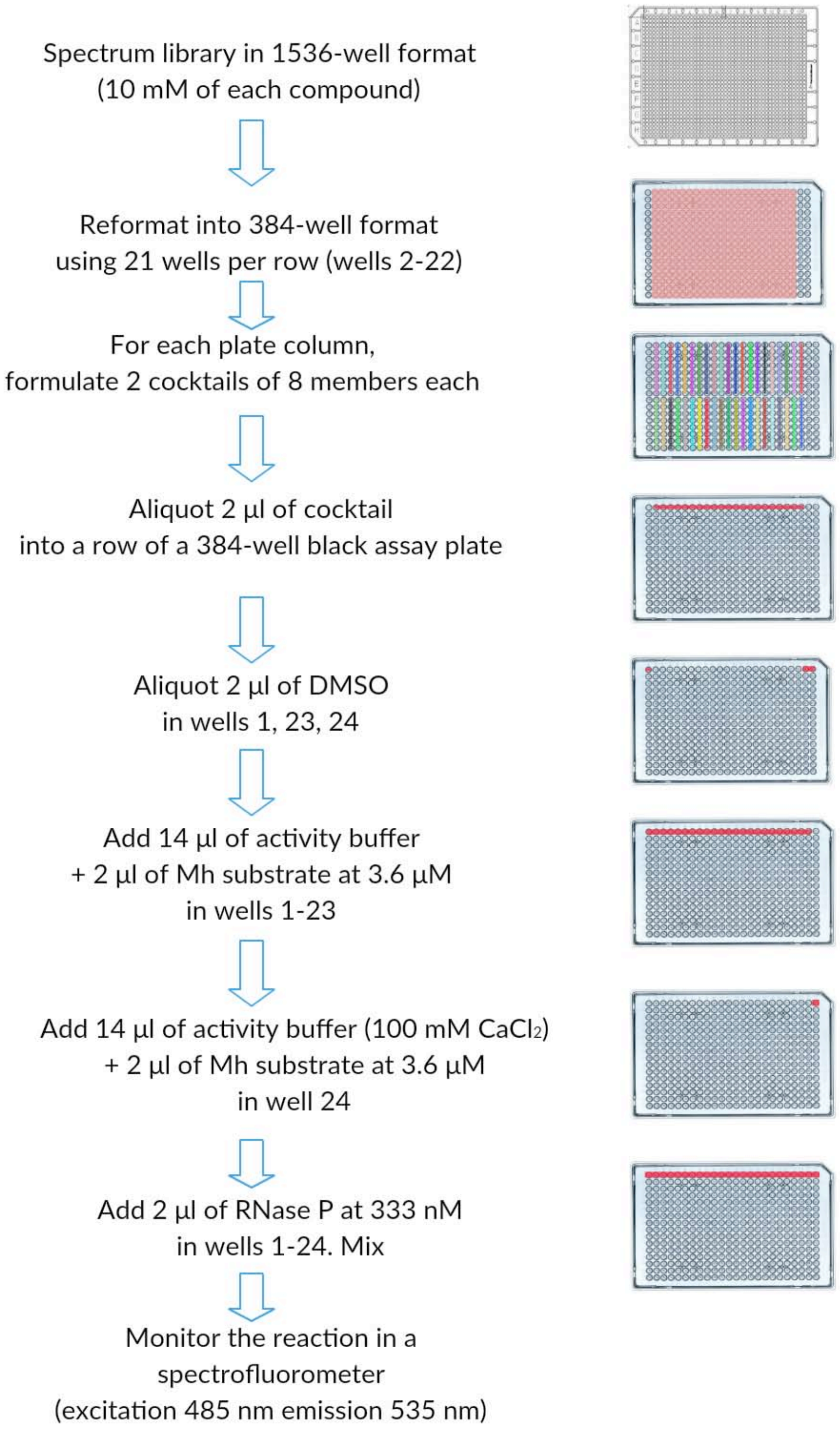

Supplementary Figure 24. Overview of the initial screening procedure using the fluorescent activity assay. See first step of Supplementary Figure 22 and Supplementary Figure 6 for the screening results. Steps 4-9 were performed using one row of the plate for each screening round.

Supplementary Table 1. Characteristics of the substrate probes used for the FRET RNase P activity assay.

	**Characteristic**	**Details / Reference**
**Fluorophore**	Alexa Fluor 488	Excitation λ_{max} = 494 nm<br>Absorbance λ_{max} = 495 nm<br>Emission λ_{max} = 517 nm (4)
**Quenchers used and their interactions with Alexa Fluor 488**	Black Hole Quencher 1 (BHQ1)	A_{max} = 534 nm<br>Q_{FRET} = 91%<br>$Q_{contact}$ = 95%<br>(4)
	Black Hole Quencher 2 (BHQ2)	A_{max} = 580 nm<br>Q_{FRET} = 91%<br>$Q_{contact}$ = 93%<br>(4)
**Fluorophore position**	BHQ at 5′ end;<br>Alexa Fluor 488 at 3′ end	Guanine at the 5′ end may have a quenching effect over fluorophore (4)
**Mh probe sequence (leader in bold)**	5′-**GAU CUG AA**U GCG GAA ACG CGC CAC-3′	(5) , (6)
**Mh size**	24 nucleotides	Molecular weight = 8,947.7 g/mol
**Mh secondary structure**	High probability of forming 3-4 bp stem	Theoretical prediction with RNA fold (7)
**bipartite pre-tRNA 5′ sequence (leader in bold)**	5′-**GAU CUG AA**G GCC AGG UAG CUC AGU UGG UAG AGC ACU GGA-3′	
**bipartite pre-tRNA 5′ size**	39 nucleotides	Molecular weight = 13,091 g/mol
**bipartite pre-tRNA 3′ sequence**	5′-UCC AGG UGU CGG CGG UUC GAU UCC GCC CCU GGC CAC CA -3′	
**bipartite pre-tRNA 3′ size**	38 nucleotides	Molecular weight = 12,806.8 g/mol

Supplementary Table 2 Data collection and refinement statistics

	TmP: (purpurin)<br>PDB code 6MAX
**Data collection**	
Space group	$I2_1$
Cell dimensions	
a, *b*, *c* (Å)	60.4, 32.7, 64.1
α, β, γ (°)	90.0, 113.2, 90.0
Resolution (Å)	51.91-1.42 (1.44-1.42) *
R_{sym} or R_{merge}	0.055 (0.394)
I/σ	11.2 (1.7)
Completeness (%)	99.7 (97.2)
Redundancy	4.7 (4.1)
Mean (I) half-set correlation $CC_{(1/2)}$	0.998 (0.886)
Wilson B-factor (Å^2)	17.2
**Refinement**	
Resolution (Å)	51.91-1.42 (1.46-1.42)
No. reflections	21010(1544)
$R_{work/}$ R_{free}	0.143/0.181 (0.249/0.308)
No. atoms	
Protein	951
Ligand/ion	19 (lig), 10 (sulfate)
Water	105
B-factors	
Protein (by chain)	26.4
Ligand/ion	42.5 (lig), 32.5 (sulfate)
Water	31.1
R.m.s deviations	
Bond lengths (Å)	0.008
Bond angles (°)	1.098

One crystal was used.
*Highest resolution shell is shown in parentheses.

Supplementary information about the selected inhibitory compounds

The Spectrum Collection (MicroSource Discovery Systems, Inc) used in this study includes biologically active compounds.

Some relevant features for each of the four selected hits regarding the inhibition of *T. maritima* RNase P include:

Crystal violet / gentian violet (Tris(4-(dimethylamino)phenyl)methylium chloride).

Although long-known to have antibacterial effects, the mechanism of action for this compound is not yet established (although multiple hypotheses exist (reviewed in (8))). Gentian violet and the chemically-related compound malachite green bind to DNA quadruplexes (9) and malachite green induces DNA damage (10). These effects correlate with the non-specific effect on RNA that we monitored in this study.

Juglone (5-hydroxy-1, 4-naphthoquinone).

This compound is extracted from the black walnut tree and has been used as an antibacterial agent. A recent study aimed to identify proteins of *Staphylococcus aureus* induced after treatment with juglone (11), (12) indicated that several proteins involved in DNA and RNA metabolism are affected. The authors suggest a possible direct interaction of juglone with nucleic acids. Our results suggest that specific interactions with some proteins are also feasible.

Purpurin (1,2,4-Trihydroxyanthraquinone). The anthraquinones are also known for their antibacterial effects. They seem to bind nucleic acids non-specifically by a groove-binding mode (13). Again, this study suggests that purpurin may specifically bind to some proteins.

Hematein. Although it is known that this compound forms complexes with aluminum that bind to DNA and RNA (14), hematein has also been described as a specific inhibitor of casein kinase 2 (CK2) (15).

REFERENCES

1. Zhang, J.H., Chung, T.D. and Oldenburg, K.R. (1999) A Simple Statistical Parameter for Use in Evaluation and Validation of High Throughput Screening Assays. *J Biomol Screen*, **4**, 67-73.
2. Buck, A.H., Dalby, A.B., Poole, A.W., Kazantsev, A.V. and Pace, N.R. (2005) Protein activation of a ribozyme: the role of bacterial RNase P protein. *EMBO J*, **24**, 3360-3368.
3. Talbot, S.J. and Altman, S. (1994) Gel retardation analysis of the interaction between C5 protein and M1 RNA in the formation of the ribonuclease P holoenzyme from Escherichia coli. *Biochemistry*, **33**, 1399-1405.

4. Marras, S.A., Kramer, F.R. and Tyagi, S. (2002) Efficiencies of fluorescence resonance energy transfer and contact-mediated quenching in oligonucleotide probes. *Nucleic Acids Res*, **30**, e122.
5. Brannvall, M., Kikovska, E., Wu, S. and Kirsebom, L.A. (2007) Evidence for induced fit in bacterial RNase P RNA-mediated cleavage. *J Mol Biol*, **372**, 1149-1164.
6. Wu, S., Chen, Y., Mao, G., Trobro, S., Kwiatkowski, M. and Kirsebom, L.A. (2014) Transition-state stabilization in Escherichia coli ribonuclease P RNA-mediated cleavage of model substrates. *Nucleic Acids Res*, **42**, 631-642.
7. Gruber, A.R., Lorenz, R., Bernhart, S.H., Neubock, R. and Hofacker, I.L. (2008) The Vienna RNA websuite. *Nucleic Acids Res*, **36**, W70-74.
8. Maley, A.M. and Arbiser, J.L. (2013) Gentian violet: a 19th century drug re-emerges in the 21st century. *Exp Dermatol*, **22**, 775-780.
9. Kong, D.M., Ma, Y.E., Wu, J. and Shen, H.X. (2009) Discrimination of G-quadruplexes from duplex and single-stranded DNAs with fluorescence and energy-transfer fluorescence spectra of crystal violet. *Chemistry*, **15**, 901-909.
10. Bose, B., Motiwale, L. and Rao, K.V. (2005) DNA damage and G2/M arrest in Syrian hamster embryo cells during Malachite green exposure are associated with elevated phosphorylation of ERK1 and JNK1. *Cancer Lett*, **230**, 260-270.
11. Wang, J., Wang, Z., Wu, R., Jiang, D., Bai, B., Tan, D., Yan, T., Sun, X., Zhang, Q. and Wu, Z. (2016) Proteomic Analysis of the Antibacterial Mechanism of Action of Juglone against Staphylococcus aureus. *Nat Prod Commun*, **11**, 825-827.
12. Wang, J., Cheng, Y., Wu, R., Jiang, D., Bai, B., Tan, D., Yan, T., Sun, X., Zhang, Q. and Wu, Z. (2016) Antibacterial Activity of Juglone against Staphylococcus aureus: From Apparent to Proteomic. *Int J Mol Sci*, **17**, 965.
13. Qiao, C., Bi, S., Sun, Y., Song, D., Zhang, H. and Zhou, W. (2008) Study of interactions of anthraquinones with DNA using ethidium bromide as a fluorescence probe. *Spectrochim Acta A Mol Biomol Spectrosc*, **70**, 136-143.
14. Bettinger, C. and Zimmermann, H.W. (1991) New investigations on hematoxylin, hematein, and hematein-aluminium complexes. II. Hematein-aluminium complexes and hemalum staining. *Histochemistry*, **96**, 215-228.
15. Hung, M.S., Xu, Z., Lin, Y.C., Mao, J.H., Yang, C.T., Chang, P.J., Jablons, D.M. and You, L. (2009) Identification of hematein as a novel inhibitor of protein kinase CK2 from a natural product library. *BMC Cancer*, **9**, 135.
